# Mesenchymal Stem Cell-Derived Exosomes Reprogram Chemosensitivity Pathways in Cervical Cancer Spheroids

**DOI:** 10.3390/ijms27031575

**Published:** 2026-02-05

**Authors:** Piyatida Molika, Kesara Nittayaboon, Kankamol Kerdkumthong, Raphatphorn Navakanitworakul

**Affiliations:** 1 Department of Biomedical Sciences and Biomedical Engineering, Faculty of Medicine, Prince of Songkla University, Hat Yai 90110, Songkhla, Thailand; piyatidamolika@gmail.com (P.M.); kesara.nittayaboon@gmail.com (K.N.); kkerdkumthong@gmail.com (K.K.); 2 Translational Medicine Research Center, Faculty of Medicine, Prince of Songkla University, Hat Yai 90110, Songkhla, Thailand

**Keywords:** exosomes, chemotherapy, spheroids, apoptosis, mesenchymal stem cells, cervical cancer, drug resistance

## Abstract

Cervical cancer (CC) remains a major global health challenge due to chemotherapy resistance and recurrence. Mesenchymal stem cell-derived exosomes (MSC-exosomes) have dual roles, as they can act as therapeutic agents and contribute to chemoresistance. However, their role in response to chemotherapy in CC remains unclear. Therefore, our study investigated the effects of MSC-exosome pretreatment on chemotherapy sensitivity using three-dimensional spheroid models generated from HeLa and SiHa CC cell lines. Proteomic profiling of MSC-exosomes identified key proteins, including ANXA1, ANXA2, EEF2, LGALS1, and PKM2, associated with tumor regeneration and chemotherapy response. MSC-exosomes exhibited context-dependent effects in both chemoresistance and chemosensitization by modulating drug efflux, metabolic reprogramming, stress adaptation, apoptosis, DNA damage response, and integrin-mediated signaling. MSC-exosome pretreatment altered spheroid responses to paclitaxel in combination with cisplatin or carboplatin. MSC-exosomes significantly enhanced chemotherapy-induced cytotoxicity in HeLa spheroids, as evidenced by reduced cell viability, increased caspase activity, and upregulation of the pro-apoptotic marker Bax. In contrast, SiHa spheroids represented selective responses: MSC-exosome pretreatment did not enhance sensitivity to paclitaxel–cisplatin but improved responsiveness to paclitaxel–carboplatin, particularly within the spheroid core. Overall, MSC-exosome pretreatment exerts cell type and drug-specific effects in CC spheroids, supporting their potential to modulate chemotherapy response.

## 1. Introduction

Cervical cancer (CC) remains one of the most prevalent gynecological malignancies worldwide and is associated with significant morbidity and mortality, particularly in advanced or recurrent disease states. Despite the availability of standard approaches like chemotherapy, radiotherapy, and targeted treatment options, there is often limited success due to the development of drug resistance, tumor relapse, and treatment-related toxicities [1,2]. The complexity of CC treatment responses, especially under conditions that mimic in vivo tumor behavior, underscores the need for advanced preclinical models and mechanistic studies.

To address these challenges, three-dimensional (3D) spheroid systems have emerged as physiologically relevant tumor models that more accurately simulate the in vivo microenvironment. Unlike traditional 2D monolayers, 3D spheroids recreate critical features of solid tumors, including cell–cell interactions, oxygen and nutrient gradients, hypoxic zones, and restricted drug diffusion. These characteristics allow for a more accurate evaluation of chemotherapeutic efficacy and treatment resistance. Moreover, growing evidence suggests that mesenchymal stem cell (MSC)-derived exosomes—small extracellular vesicles involved in intercellular communication—play dual and context-dependent roles in cancer progression, metastasis, and drug resistance. Their ability to modulate immune responses, alter signaling pathways, and transfer bioactive proteins positions them as both potential therapeutic agents and contributors to chemoresistance [3,4,5]. However, their influence on CC chemotherapy responses remains insufficiently understood.

This study selected HeLa and SiHa cell lines as representative CC models due to their distinct biological and molecular profiles. HeLa cells, derived from adenocarcinoma and harboring HPV18, are characterized by high proliferative capacity, elevated anti-apoptotic signaling, and compact spheroid formation in 3D culture. In contrast, SiHa cells originate from squamous cell carcinoma, carry HPV16, and exhibit a more aggressive phenotype with greater intrinsic resistance to chemotherapy, which is partially mediated by enhanced oxidative stress responses and robust DNA repair mechanisms [6,7]. These differences make the two cell lines suitable models for comparative assessments of chemoresistance, spheroid architecture, and tumor biology [8,9]. Supporting this rationale, our previous analyses found that HeLa spheroids displayed elevated cadherin and actin expression, thereby forming dense structures that could impede drug penetration. Meanwhile, although SiHa spheroids expressed similar adhesion molecules, they failed to develop equally compact architecture; this reveals fundamental differences in their cell–cell interaction dynamics [10]. Such contrasting structural characteristics highlight the relevance of using 3D spheroid models to explore mechanisms that influence drug permeability and treatment outcomes.

Building on proteomic profiling of MSC-derived exosomes performed via liquid chromatography–tandem mass spectrometry (LC-MS/MS), we identified numerous exosomal proteins associated with chemotherapy-related pathways. Importantly, a subset of these proteins demonstrated potential roles in modulating chemosensitivity, thereby suggesting that MSC-exosomes may deliver molecular cargo capable of enhancing or dampening therapeutic responses. Since platinum-based agents (e.g., cisplatin and carboplatin) and paclitaxel remain the backbone of CC chemotherapy, understanding how exosome-mediated signaling interacts with these drugs is essential. Cisplatin induces DNA cross-linking, while paclitaxel disrupts microtubule dynamics; however, the dense structure of spheroids can limit their distribution and effectiveness [11,12].

**Figure 1:**
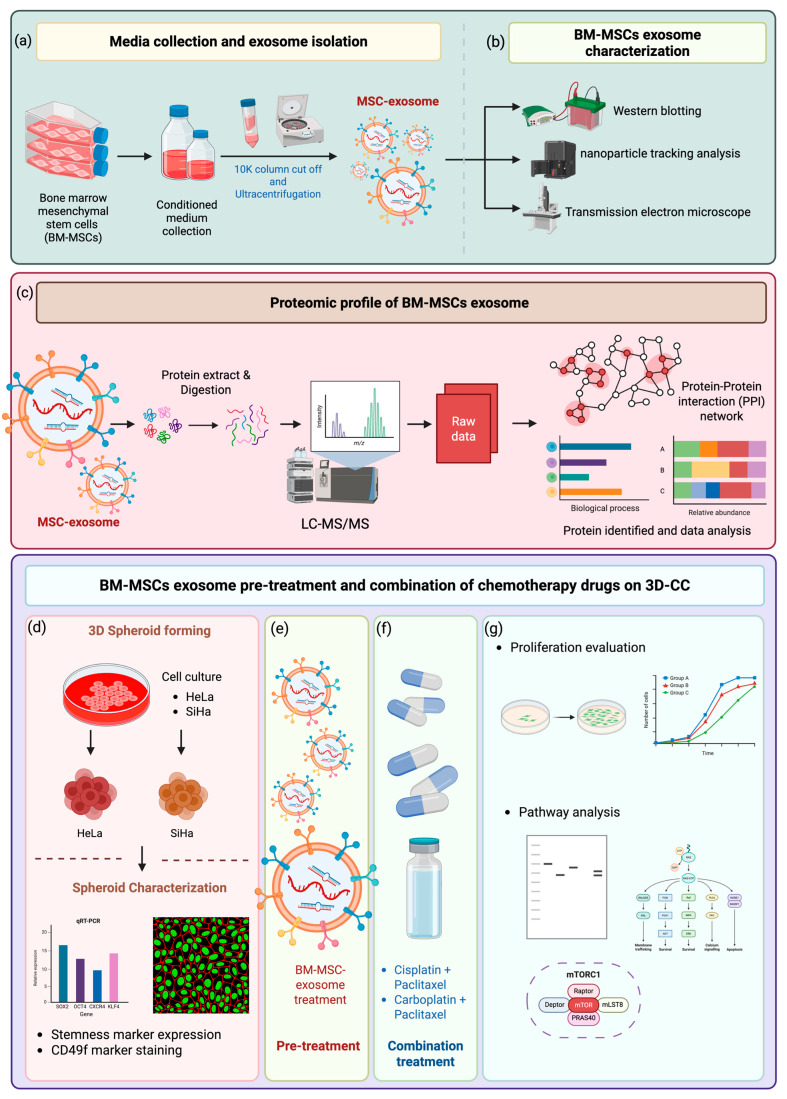
Schematic workflow for studying the effects of MSC-exosome pretreatment on combination chemotherapy in 3D cervical cancer (CC) cell models. (**a**) Mesenchymal stem cells (MSCs) were cultured to collect conditioned medium. MSC-derived exosomes were isolated using 10K Amicon filtration, followed by ultracentrifugation. (**b**) The exosome pellets were characterized using Western blotting, nanoparticle tracking analysis (NTA), and transmission electron microscopy (TEM). (**c**) Proteomic profiling of MSC-exosomes was performed using LC-MS/MS, followed by protein identification and biological process annotation. (**d**) CC cell lines, HeLa and SiHa, were cultured and induced to form 3D spheroids in poly-2-hydroxyethyl methacrylate (HEMA)-coated U-bottom plates. (**e**) MSC-exosomes were added to the spheroids as a 24 h pretreatment. (**f**) Following pretreatment, doublet chemotherapy, paclitaxel combined with either cisplatin or carboplatin, was administered for 72 h. (**g**) After treatment, cell viability assays and pathway analyses were conducted to assess therapeutic responses and molecular changes.

Therefore, this study aimed to investigate whether MSC-exosome pretreatment alters the response of HeLa and SiHa 3D spheroids to combination chemotherapy. We analyzed the protein cargo of MSC-derived exosomes, evaluated their interactions with chemotherapeutic agents, and assessed whether exosomes enhance or suppress apoptosis in these spheroid models. This work integrates molecular and functional analyses to clarify the context-dependent effects of MSC-exosomes on CC treatment and inform more personalized therapeutic strategies. The experimental workflow is summarized in Figure 1.

## 2. Results

### 2.1. Spheroid Formation and Characterization

**Figure 2:**
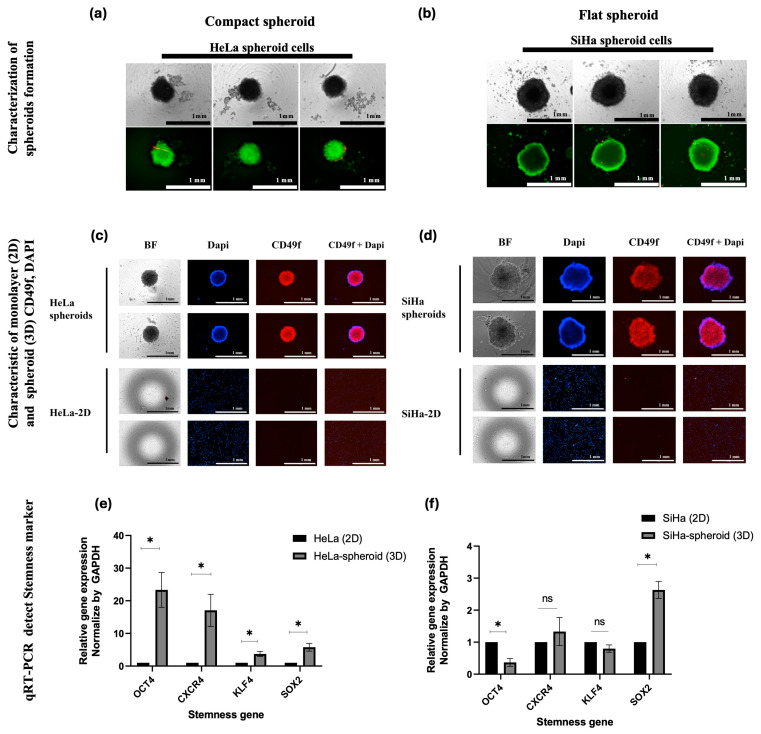
Characterization of CC spheroids. (**a**,**b**) Fluorescence images of HeLa and SiHa spheroids stained using the live/dead assay after their respective formation periods (HeLa: 3 days; SiHa: 7 days). Live cells stained green (calcein AM); dead cells stained red (ethidium homodimer-1). (**c**,**d**) Immunofluorescence staining for CD49f (integrin α6), a cancer stem cell marker, in spheroids and 2D cultures. Nuclei were counterstained with DAPI (blue). (**e**,**f**) Relative mRNA expression of stemness markers (OCT4, CXCR4, KLF4, and SOX2), measured using qRT-PCR. Expression was normalized to that of *GAPDH*, and fold change was calculated relative to 2D cultures. Data represent three independent experiments. Scale bars: 1 mm for live/dead assay, bright field (BF), 4′,6-diamidino-2-phenylindole dihydrochloride (DAPI), CD49f, and merged images. For qRT-PCR, error bars represent mean ± SD. Data are presented as mean ± SD. * *p* < 0.05; ns, not significant.

HeLa and SiHa CC cell lines were used to generate the 3D spheroid models. Spheroids were formed using in-house poly-2-hydroxyethyl methacrylate (HEMA)-coated plates, with HeLa spheroids forming within 3 days and SiHa spheroids requiring 7 days. Upon full formation, distinct morphological differences were observed between the two: HeLa spheroids were compact and rounded, whereas SiHa spheroids exhibited a flatter, less cohesive structure (Figure 2a,b). Further, HeLa spheroids were slightly smaller than SiHa spheroids. Both spheroid types maintained high viability and lacked necrotic cores, indicating the preservation of nutrient and oxygen gradients typical of 3D tumor architecture. Additionally, compared to their respective 2D cultures, both spheroid types exhibited an elevated expression of CD49f, which suggests an enrichment of a cancer stem cell (CSC)-like population in the 3D models (Figure 2c,d). CD49f, also known as integrin α6, is a surface adhesion molecule widely used as a stem cell marker. Its upregulation in spheroids may support CSCs-associated properties such as enhanced extracellular matrix (ECM) attachment, increased signaling activity, and a potential role in chemoresistance and tumor aggressiveness.

To further characterize the stem-like features, the expression of stemness-related genes, including octamer-binding transcription factor 4 (OCT4), C-X-C chemokine receptor type 4 (CXCR4), Krüppel-like factor 4 (KLF4), and SRY-box transcription factor 2 (SOX2), was assessed using quantitative reverse transcription PCR (qRT-PCR). All four genes were significantly more upregulated in HeLa spheroids than in the 2D monolayer culture. In contrast, SiHa spheroids exhibited significant upregulation of SOX2 only, while CXCR4 and KLF4 levels remained unchanged. Notably, OCT4 expression was more downregulated in SiHa spheroids than in monolayer cultures (Figure 2e,f). These findings suggest that the regulation of stemness-related genes in 3D cultures is complex and may be influenced by spheroid architecture and microenvironmental conditions that differentially modulate CSC pathways. Despite differences in gene expression patterns, both HeLa and SiHa spheroids demonstrated stemness features, reinforcing the inherent heterogeneity of CC subtypes and their distinct CSC-associated behaviors.

The IC50 values for cisplatin and carboplatin were determined using dose–response assays. Marked differences in drug sensitivity were observed between the two spheroid models (Table 1, Figure S1). Notably, compared to HeLa spheroids, SiHa spheroids showed approximately three-fold higher IC50 values for both drugs, indicating a significantly higher degree of chemoresistance. Specifically, the IC50 values in HeLa spheroids were 6.05 ± 0.52 and 49.09 ± 0.61 µg/mL for cisplatin and carboplatin, respectively. In contrast, SiHa spheroids showed significantly higher IC50 values (17.45 ± 0.49 and 157.01 ± 10.03 µg/mL, respectively).

### 2.2. Characterization of MSC-Exosomes

**Figure 3:**
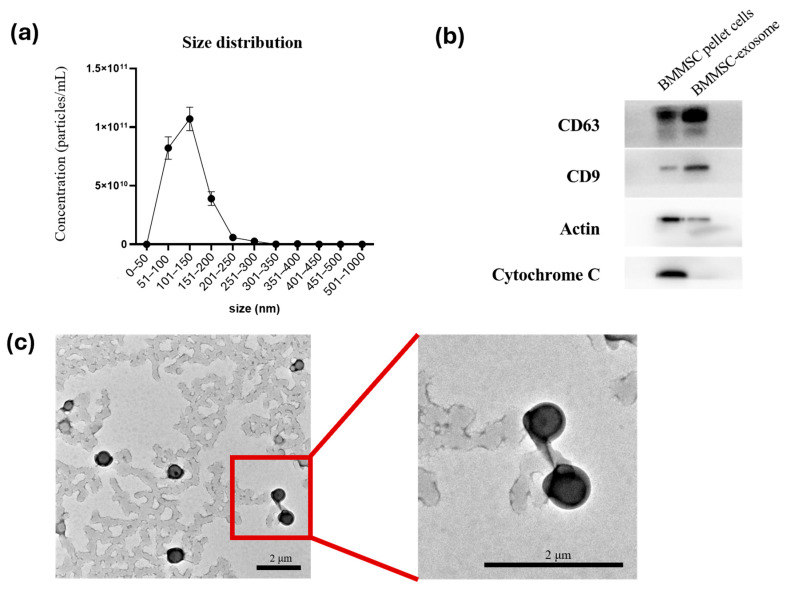
Characterization of MSC-derived exosomes isolated from conditioned medium. (**a**) NTA was used to determine the total particle count and size distribution of isolated exosomes. (**b**) Western blot analysis of MSC-derived exosome pellets (20 µL loaded per well) was performed to detect exosomal surface markers CD63 and CD9. β-actin was used as an internal control, and cytochrome C was included as a negative marker to confirm the purity of the exosomal preparation. (**c**) TEM revealed the typical cup-shaped morphology of MSC-exosomes.

MSC-derived exosomes were isolated from conditioned mediums according to concentration using a 10 kDa Amicon Ultra centrifugal filter, followed by purification with qEV size-exclusion chromatography and ultracentrifugation. Thereafter, the obtained exosomes were characterized using nanoparticle tracking analysis (NTA), Western blotting, and transmission electron microscopy (TEM) to confirm their size distribution, morphology, and surface marker expression. NTA results revealed that the majority of exosomes ranged in size from 100 to 250 nm (Figure 3a). To assess the purity of the extracellular vesicles (EVs) and exclude contamination from the culture medium, Western blotting was performed for canonical exosomal markers, including CD63 and CD9, with β-actin used as a loading control. Both CD63 and CD9 were detected in the MSC-exosome fractions (Figure 3b), confirming the identity of the isolated vesicles. TEM analysis was performed to visualize the morphology, structure, and size of the exosomes. TEM images showed the typical cup-shaped morphology characteristic of exosomes (Figure 3c).

Collectively, these data confirmed the successful isolation and characterization of MSC-derived exosomes from the conditioned medium, which were subsequently stored for further experiments.

### 2.3. Proteomic Profiling and Enrichment Analysis of MSC-Exosome Proteins

**Figure 4:**
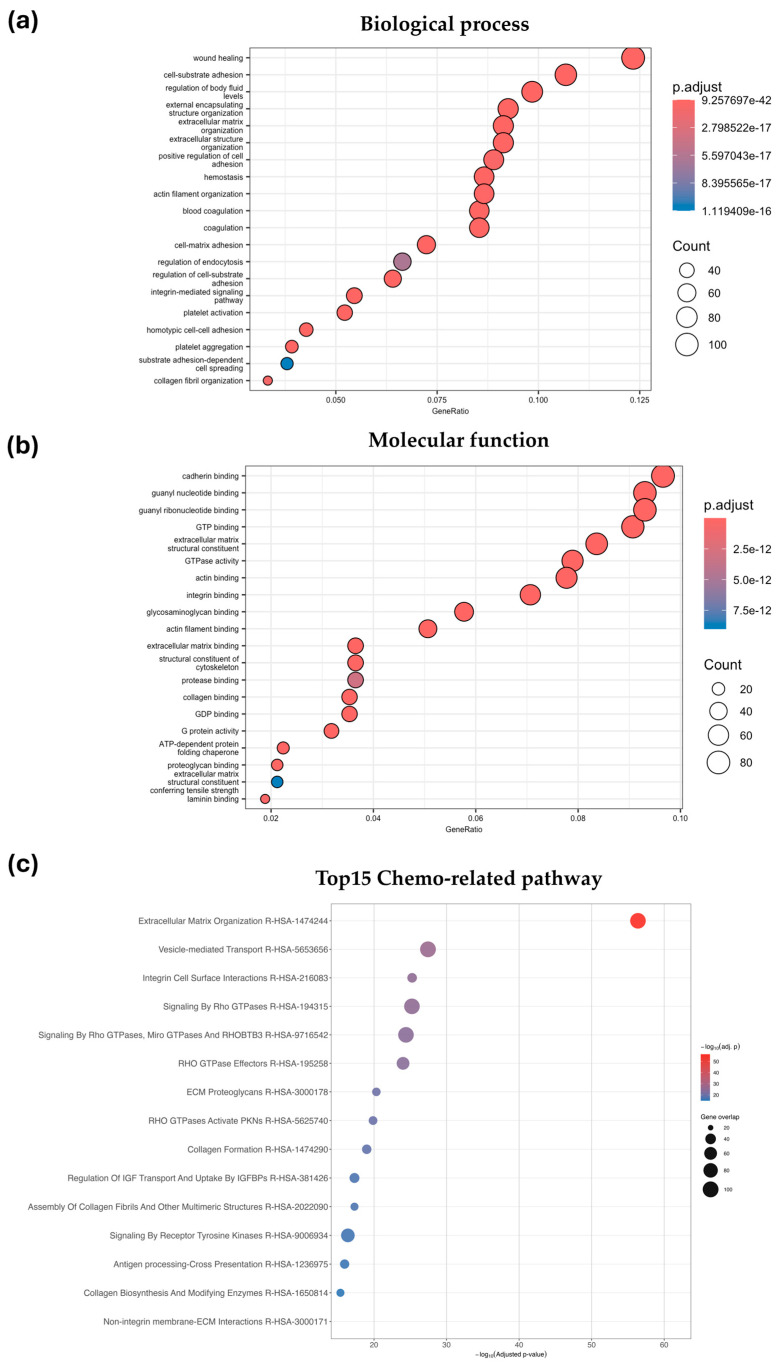
Gene ontology (GO) and pathway enrichment analysis of MSC-exosome proteins: (**a**) biological process; and (**b**) molecular function categories associated with the identified proteins, performed using the PANTHER database (Version 19.0). The *x*-axis represents the GeneRatio (proportion of proteins in each category), while the *y*-axis lists the corresponding GO terms. (**c**) Bubble plot of top 15 chemotherapy-related pathways identified from MSC-exosome proteomic profiling based on the Reactome database (Version 95.0). The *x*-axis shows statistical significance (–log_10_ adjusted *p*-value), and the *y*-axis lists enriched pathways. Bubble size corresponds to the number of overlapping proteins in each pathway, while bubble color indicates enrichment significance.

LC–MS/MS was used to characterize the protein profiles of MSC-derived exosomes. A total of 787 proteins were identified following isolation and purification, and enrichment analyses of biological processes (BP) and molecular functions (MF) were performed. Gene ontology (GO) biological process enrichment analysis demonstrated that proteins identified in MSC-exosomes were predominantly associated with ECM organization, cell adhesion, and tissue remodeling (Figure 4a). Notably, wound healing was the most enriched biological process among the mapped gene set. Processes related to cell–ECM interactions, including cell-substrate adhesion, cell-matrix adhesion, and extracellular matrix organization, were highly represented. In addition, hemostasis-related processes, such as hemostasis, blood coagulation, and coagulation, were significantly enriched. Detection of cytoskeleton-associated processes, including actin filament organization, further suggest the role of MSC-exosome proteins in regulating cellular structural dynamics. Collectively, these findings indicate that a substantial proportion of proteins detected in MSC-exosomes are functionally associated with ECM remodeling, adhesion signaling, and tissue repair-related biological processes. Molecular function enrichment analysis revealed that MSC-exosome proteins were generally involved in binding and structural activities (Figure 4b). Among the significantly enriched molecular functions, cadherin binding, integrin binding, and actin binding were consistently identified. Functions associated with cytoskeletal and extracellular matrix structure, including extracellular matrix structural constituent and structural constituent of cytoskeleton, were also prominently enriched. Moreover, signaling-related functions such as GTP binding, GDP binding, and GTPase activity were also observed, which suggests the involvement of MSC-exosome proteins in small GTPase-mediated signaling pathways. Collectively, these data indicate that a considerable proportion of MSC-exosome proteins possess molecular functions critical for adhesion, cytoskeletal regulation, and intracellular signaling. Overall, GO enrichment analysis revealed that MSC-exosome-associated proteins are involved in key biological processes and molecular functions related to extracellular matrix organization, adhesion, and cytoskeletal dynamics.

Pathway enrichment analysis performed to explore potential chemotherapy modulation identified the top 15 chemotherapy-related pathways associated with MSC-exosome proteins. Extracellular matrix organization was the most enriched pathway, followed by integrin-mediated signaling, and Rho GTPase signaling, thereby highlighting pathways involved in adhesion, migration, and cytoskeletal dynamics (Figure 4c). Overall, MSC-exosome proteins were enriched in processes supporting core cellular functions (binding, catalytic activity, metabolism, and regulation) along with adaptive responses including stress response, transporter activity, and antioxidant defense. The integrated BP and MF profiles suggest that MSC-exosome protein cargo may contribute to the modulation of drug response, stress signaling, and chemotherapy-related mechanisms.

### 2.4. STRING Enrichment Network of MSC-Exosome Proteins

**Figure 5:**
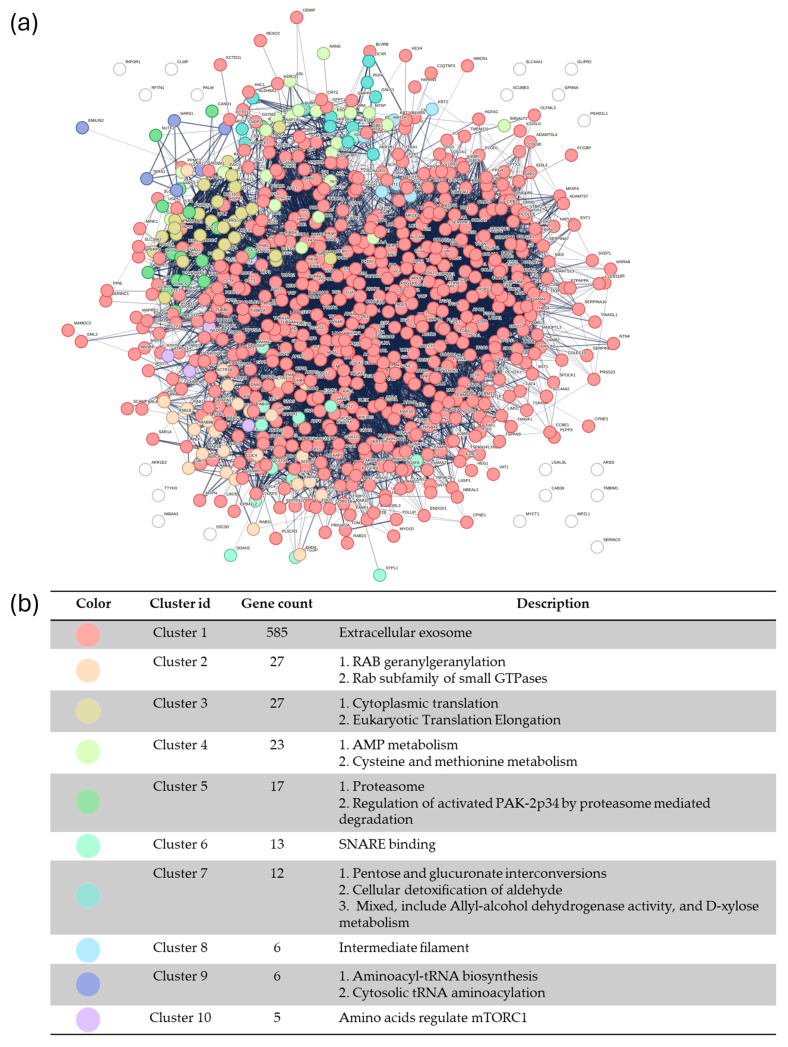
STRING protein–protein interaction (PPI) network of MSC-exosome proteins. (**a**) The network shows functional associations among identified proteins. Nodes represent proteins; edges indicate predicted or validated interactions. Colored clusters represent functionally enriched groups, while white nodes lack significant interactions. (**b**) Functional clustering table summarizes the top 10 clusters, color-coded to match the network, including extracellular exosome (Cluster 1), Rab GTPase processes (Cluster 2), cytoplasmic translation (Cluster 3), AMP metabolism (Cluster 4), proteasome/protein degradation (Cluster 5), SNARE binding (Cluster 6), and other clusters such as intermediate filament organization and mTORC1 regulation.

Protein–protein interaction (PPI) network analysis of proteins identified in MSC-derived exosomes revealed a highly interconnected network, which indicates extensive functional coordination among the detected proteins (Figure 5). Network clustering analysis identified 10 distinct functional clusters. Cluster 1 was the dominant module comprising 585 proteins and was strongly enriched for extracellular exosome-associated proteins, thereby supporting the vesicle-associated nature of the dataset. Several additional clusters with distinct functional themes were also observed. Cluster 2 was enriched in pathways related to Rab geranylgeranylation and Rab subfamily small GTPases, thus highlighting processes involved in vesicle trafficking and membrane dynamics. Cluster 3 (27 proteins) comprised primarily of proteins associated with cytoplasmic translation and eukaryotic translation elongation, whereas Cluster 4 was enriched for pathways related to AMP metabolism and cysteine and methionine metabolism, thereby suggesting metabolic regulation within the detected protein cargo. Proteostasis-related pathways were represented by Cluster 5, which was enriched for proteasome-associated processes including the regulation of activated PAK-2p34 degradation. Additional clusters included SNARE binding (Cluster 6), detoxification and carbohydrate metabolism pathways such as pentose and glucuronate interconversions (Cluster 7), structural or regulatory modules related to intermediate filaments (Cluster 8), aminoacyl-tRNA biosynthesis (Cluster 9), and amino acid-mediated regulation of mTORC1 signaling (Cluster 10). Collectively, this network analysis demonstrates that proteins identified in MSC-derived exosomes form a complex and functionally diverse interaction network encompassing pathways involved in vesicle trafficking, protein synthesis, metabolic regulation, and intracellular signaling.

Further examination of the STRING clusters revealed several functional modules associated with biological processes relevant to chemotherapy response. Cluster 1 reflects vesicle-associated proteins potentially involved in cargo delivery and modulation of drug sensitivity. Meanwhile, Cluster 2 (Rab GTPase family) is linked to vesicle trafficking and drug efflux mechanisms implicated in chemoresistance. Additionally, Cluster 3 (cytoplasmic translation/elongation) and Cluster 4 (AMP, cysteine, and methionine metabolism) are associated with translational stress and redox balance, processes critical for cell survival under platinum-based chemotherapy. Cluster 5 (proteasome and PAK2-p34 degradation) involves protein turnover and apoptotic signaling, which are relevant to paclitaxel response. Cluster 6 (SNARE binding) highlights vesicle fusion machinery that may influence drug delivery and intercellular communication. Cluster 7 (detoxification and pentose/gluconate metabolism) is linked to metabolic reprogramming and oxidative stress buffering. Smaller clusters, such as Cluster 9 (tRNA biosynthesis) and Cluster 10 (amino acid-mediated regulation of mTORC1), emphasize translational control and mTOR signaling pathways that can influence both drug sensitivity and resistance.

Overall, the STRING clustering analysis indicates that proteins identified in MSC-derived exosomes are enriched in functional modules related to drug metabolism, stress response, apoptosis regulation, and intracellular signaling. These findings support the potential involvement of MSC-exosome protein cargo in modulating therapeutic responses in CC, while acknowledging that these associations are based on bioinformatic enrichment analyses.

### 2.5. Functional Annotation and Chemotherapy-Relevant Pathway Analysis of MSC-Exosome Proteins

Proteomic analysis of MSC-derived exosomes revealed numerous biological processes relevant to chemotherapy response. Drug-related mechanisms, including ABC transporter-mediated drug efflux (ABCC1, ABCC4) and detoxification via glutathione metabolism (GSTP1, GSTT1, GSR, GPX3/PRDX1/2/4/6, TXNL1), were well represented (Table 2). Metabolic regulators included aldo–keto reductases and aldehyde dehydrogenases (AKR1B1, AKR1B10, AKR1C1/3, ALDH1A1/ALDH9A1), glycolytic and Warburg effect enzymes (PKM2, PGK1, LDHA/LDHB, ENO1, PFKP, TPI1, PGAM1, ALDOA, ALDOC, FASN, ACLY), pentose phosphate pathway enzymes (G6PD, PGD, TALDO1, TKT), and one-carbon/folate metabolism proteins (DHFR, MTHFD1, ALDH1L1). Transport processes included lactate export (SLC16A3/MCT4) and amino acid uptake supporting mTOR signaling (SLC7A5, SLC1A5). Cytoskeletal and epithelial–mesenchymal transition (EMT)-associated proteins (VIM, FLNA, FSCN1, ACTN1/4, IQGAP1, EZR, TAGLN) and integrin/adhesion components (ITGA1–6, ITGAV, ITGB1/2/3/5, ILK, TLN1, LIMS1, VCL, ZYX) were abundant, alongside ECM regulators (FN1, COL family, SPARC, POSTN, TNC, THBS1/2/4, LOX, VCAN, TGFB1). Key signaling pathways included RTK/MAPK/PI3K–AKT (MAPK1, PDGFRB, PRKCB, YES1, LYN, PIK3CG, STAT1) and Ras/Rho GTPases (KRAS, NRAS, RAC1, CDC42, ARHGDIA, RALA, RALB). Proteostasis and stress responses were reflected by chaperones and proteasome components (HSP90, HSPA family, VCP, UBA1, UBE2, PSMA/PSMC/PSMD), unfolded protein response/ER stress markers (HSPA5, PDIA3, HSP90B1), and autophagy/mitophagy proteins (MAP1LC3A/B, DNM1L). Proteins involved in exosome biogenesis and trafficking (RAB family, SNAP23, STX4/7, STXBP2/3, TSG101, ALIX, SCAMP3, TOM1) were also prominent. Moreover, apoptosis regulators (ANXA1/2, LGALS1, EEF2) and signaling molecules of the TGF-β/Notch (TGFB1, NOTCH2/3), caveolae (CAV1, CAVIN1), and vault transport (MVP) pathways were also identified (Table 2).

Furthermore, within the identified proteins, we found several groups of proteins that can be distinguished based on their roles in stress adaptation, redox balance, immune modulation, and tissue repair (in Table 2 (gray label) below). The heat shock proteins (HSPs) such as HSP90AA1, HSP90AB1, HSP90B1, HSPA1A/B, HSPA5, HSPA8, HSPB1, HSPB6, HSPA2, and HSPA4 function as molecular chaperones that safeguard against protein misfolding and enhance cell survival under adverse conditions. In terms of oxidative stress, enzymes including peroxiredoxins (PRDX1, PRDX2, PRDX4, PRDX6), thioredoxin-like protein (TXNL1), glutathione S-transferases (GSTP1, GSTT1/2, GSTM2), glutathione peroxidase (GPX3), cytochrome b5 reductase (CYB5R3), and biliverdin reductases (BLVRA, BLVRB) play essential roles in the detoxification of reactive oxygen species (ROS). Additionally, metabolic enzymes such as G6PD, PGD, IDH1, and PHGDH contribute to antioxidant defense by generating NADPH and preserving cellular redox equilibrium. Beyond oxidative protection, several proteins are linked to anti-inflammatory and immunomodulatory activities. These include annexins (ANXA1, ANXA2, ANXA5, ANXA6), which act as pro-resolving factors of inflammation, along with TGFB1, S100 proteins (S100A6, S100A10, S100A11), and AHSG (fetuin-A), all of which help to regulate inflammatory pathways and limit tissue damage. Moreover, proteins associated with the ECM play crucial roles in tissue repair and wound healing processes. Key examples include fibronectin (FN1), vitronectin (VTN), SPARC, decorin (DCN), tenascin-C (TNC), and a range of collagens (COL1A1, COL1A2, COL3A1, COL4A1/2, COL5A1/2/3, COL6A1–3, COL18A1). Likewise, laminins (LAMA1/2/4, LAMB1/2, LAMC1) and integrins (ITGB1, ITGB3, ITGB5, ITGA5, ITGA6), together with thrombospondins (THBS1, THBS2, THBS4), coordinate ECM remodeling, cell adhesion, migration, angiogenesis, and overall processes of tissue regeneration. Therefore, these findings suggest that certain proteins derived from MSC-exosome may serve as potential mediators of chemosensitization, thereby enhancing the effectiveness of chemotherapy in a cell type-specific context.

### 2.6. Exosome Uptake and Dose Optimization in CC Cells

**Figure 6:**
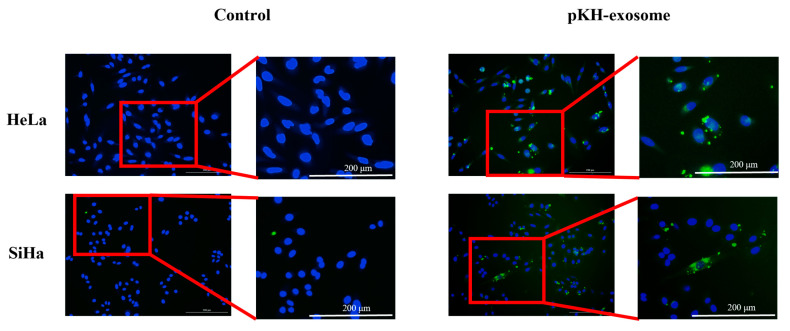
Uptake of MSC-exosomes by CC cells. PKH67-labeled MSC-exosomes (green fluorescence) were incubated with HeLa and SiHa cells under 2D culture conditions for 24 h. Subsequently, cells were fixed and counterstained with DAPI (blue) to visualize nuclei. Fluorescence microscopy showed punctate green fluorescence within the cytoplasm, which indicated successful internalization of MSC-exosomes by both CC cell lines. Scale bar: 200 μm.

Assessing exosome uptake by cancer cells is essential for verifying their biological activity, particularly before linking them to changes in gene expression or therapeutic responses. To confirm the internalization of MSC-derived exosomes by CC cells, we conducted an uptake assay using PKH67, a green, fluorescent membrane dye, to label exosome membranes. The labeled MSC-exosomes were incubated with monolayer cultures of HeLa and SiHa cells for 24 h. Exosome uptake was visualized using a live-cell imaging microscope (Agilent Technologies, Santa Clara, CA, USA). Green fluorescence enabled tracking of exosome localization, while 4′,6-diamidino-2-phenylindole dihydrochloride (DAPI) counterstaining allowed clear distinction of nuclei and helped differentiate internalized vesicles from those merely attached to the cell surface. Exosomes are typically taken up by recipient cells through endocytosis, followed by intracellular cargo release. As shown in Figure 6, most of the exosomes were observed near the nuclear region, with additional dispersion throughout the cytoplasm in both HeLa and SiHa cells. These results confirm the efficient uptake of MSC-derived exosomes by both cell lines, supporting their potential to modulate downstream cellular responses.

### 2.7. Effect of MSC-Exosome Pretreatment on Combination Chemotherapy in CC Spheroids

Based on the exosomal proteomics and enrichment analyses, MSC-exosomes were enriched in proteins associated with stress response, metabolic regulation, redox balance, apoptosis-related processes, and survival-associated signaling. These functional categories are known to influence chemotherapy response in cancer cells. Therefore, to mechanistically validate the effects of MSC-exosome pretreatment on CC spheroids, we next evaluated spheroid viability, caspase-3/7 activation, and key regulators of the DNA damage response and apoptosis pathways. Although NF-κB-related proteins were not directly enriched in the proteomics dataset, NF-κB signaling was examined due to its established role in chemoresistance and stress-mediated survival in cervical cancer.

To investigate the modulatory effects of MSC-exosome on the response to combination chemotherapy in CC spheroids, we assessed the viability of HeLa and SiHa spheroids following MSC-exosome pretreatment and subsequent exposure to combination chemotherapy. Previous studies have shown that MSC-derived exosomes can exert dual roles in cancer progression by delivering bioactive molecules that regulate apoptosis, influence proliferation pathways, and either enhance drug sensitivity or promote resistance. In this study, CC spheroids were pretreated with MSC-exosome to simulate cancer–stromal interactions and then treated with paclitaxel in combination with either cisplatin or carboplatin, that are standard regimens in CC therapy. Treatment outcomes were assessed using live/dead-cell imaging (Agilent Technologies, Santa Clara, CA, USA) and the ApoLive-GloTM multiplex assay (Promega Corporation, Madison, WI, USA), which enables us to evaluate cell viability, cytotoxicity, and caspase activity in 3D spheroid models. This approach allowed us to determine whether MSC-exosome pretreatment modulates chemotherapy response and to compare the effects across different CC cell lines.

**Figure 7:**
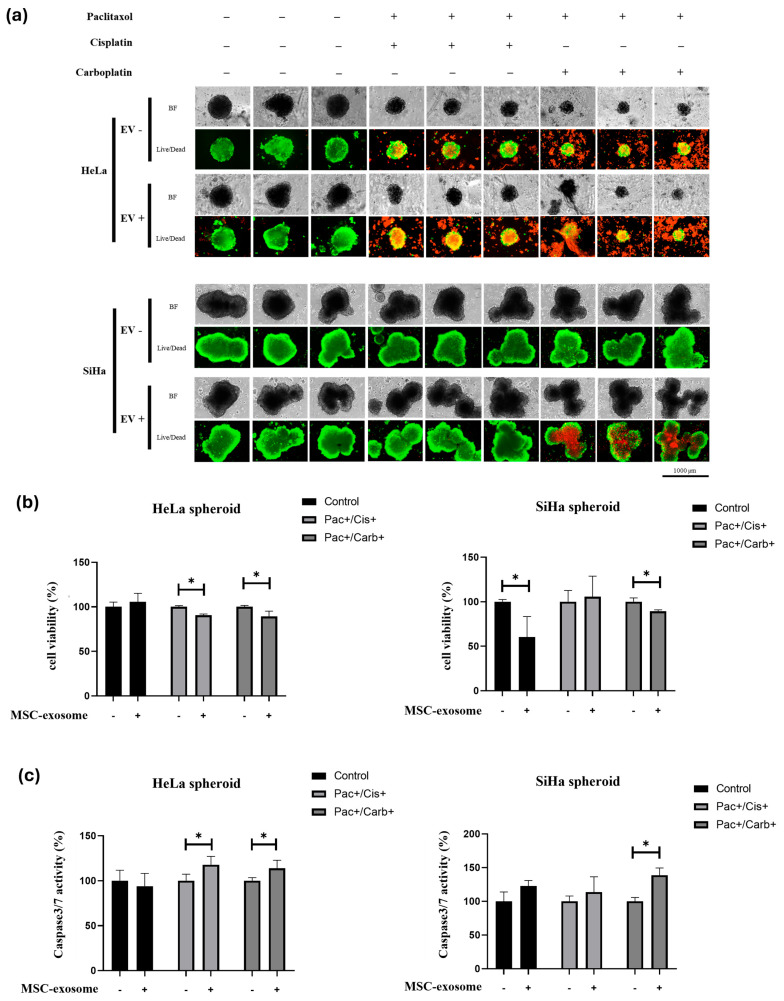
Assessment of cell viability and caspase-3/7 activity in CC spheroids following MSC-exosome pretreatment and combination chemotherapy. (**a**) HeLa and SiHa spheroids were pretreated with MSC-exosomes for 24 h, followed by treatment with paclitaxel combined with either cisplatin or carboplatin. After 48 h of drug incubation, spheroids were stained using a live/dead assay, where green fluorescence indicates viable cells and red/orange fluorescence indicates dead or membrane-compromised cells. Increased red fluorescence corresponds to reduced cell viability. (**b**) Quantification of cell viability and (**c**) caspase-3/7 activity was performed using the ApoLive-Glo™ multiplex assay. Results represent the mean ± SD of three independent experiments. Statistical significance was determined using Student’s *t*-test (* *p*-value < 0.05).

In HeLa spheroids, treatment with MSC-exosome alone did not lead to significant changes in viability compared to that in the untreated control. However, when combined with chemotherapy, MSC-exosome pretreatment induced a noticeable increase in cell death, as evidenced by enhanced red fluorescence in the live/dead assay (Figure 7a). Notably, the combination of paclitaxel and carboplatin was more effective than paclitaxel or cisplatin alone, resulting in smaller spheroids and more extensive cell death. Yellow fluorescence, resulting from overlapping green (live) and red (dead) signals, was observed in exosome-pretreated spheroids, thereby suggesting areas of mixed cell viability. These observations were consistent with the ApoLive-Glo™ multiplex assay, which revealed a significant reduction in cell viability and a corresponding increase in caspase-3/7 activity following MSC-exosome pretreatment in both combination chemotherapy groups (Figure 7b, left panel). These results indicate enhanced apoptotic signaling and reduced cell survival in HeLa spheroids (Figure 7c, left panel).

In contrast, SiHa spheroids showed a more selective response. Pretreatment with MSC-exosome alone did not produce detectable changes in the live/dead assay, with the spheroid mass remaining largely green—similar to the untreated controls. However, quantitative analysis from the ApoLive-Glo™ multiplex assay revealed a significant decrease in cell viability in the MSC-exosome-alone group (Figure 7b, right panel), thereby suggesting subtle cytotoxic effects not visible in fluorescence imaging. Treatment with paclitaxel/cisplatin, with or without MSC-exosome pretreatment, did not elicit notable cytotoxic effects in SiHa spheroids. However, a pronounced response was observed in SiHa spheroids pretreated with MSC-exosome, followed by paclitaxel and carboplatin. Strong red fluorescence appeared in the spheroid core in this group, thereby suggesting enhanced cell death compared to that in the MSC-exosome-negative group (Figure 5a). This finding was further supported by a significant reduction in cell viability and increased caspase-3/7 activity, as measured using the ApoLive-Glo™ assay (Figure 7b,c; right panel).

Collectively, these findings demonstrate that the effect of MSC-exosome pretreatment on chemotherapy sensitivity is both cell line-specific and drug-combination-dependent. MSC-exosome enhanced the efficacy of both paclitaxel/cisplatin and paclitaxel/carboplatin regimens in HeLa spheroids, which was reflected by reduced viability and increased apoptosis. In contrast, SiHa spheroids, characterized by greater intrinsic chemoresistance, exhibited improved sensitivity only when pretreated with MSC-exosome prior to paclitaxel and carboplatin exposure.

### 2.8. MSC-Exosome-Induced Alterations in Protein Expression Following Combination Chemotherapy in CC Spheroids

**Figure 8:**
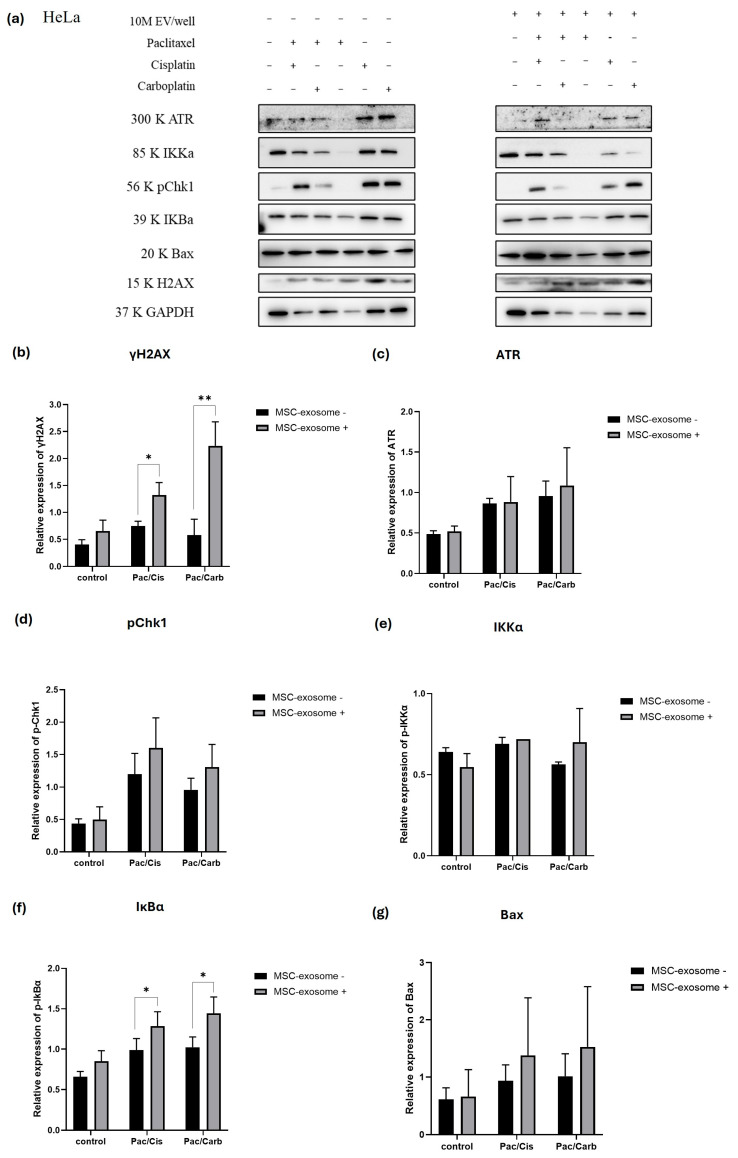
Western blot analysis of DNA damage, NF-κB, and apoptosis pathway proteins in HeLa spheroids following MSC-exosome pretreatment and combination chemotherapy. (**a**) Representative Western blot image showing the expression of key regulatory proteins in HeLa spheroids across different treatment conditions: untreated control, MSC-exosome pretreatment alone, and paclitaxel with either cisplatin or carboplatin, with or without MSC-exosome pretreatment. Densitometric analyses of the following proteins are presented as bar graphs: (**b**) γH2AX, (**c**) ATR, (**d**) pChk1, (**e**) IKKα, (**f**) IκBα, and (**g**) Bax. All protein levels were normalized to those of GAPDH. Statistical significance was determined using Student’s *t*-test (* *p*-value < 0.05, ** *p*-value < 0.01).

**Figure 9:**
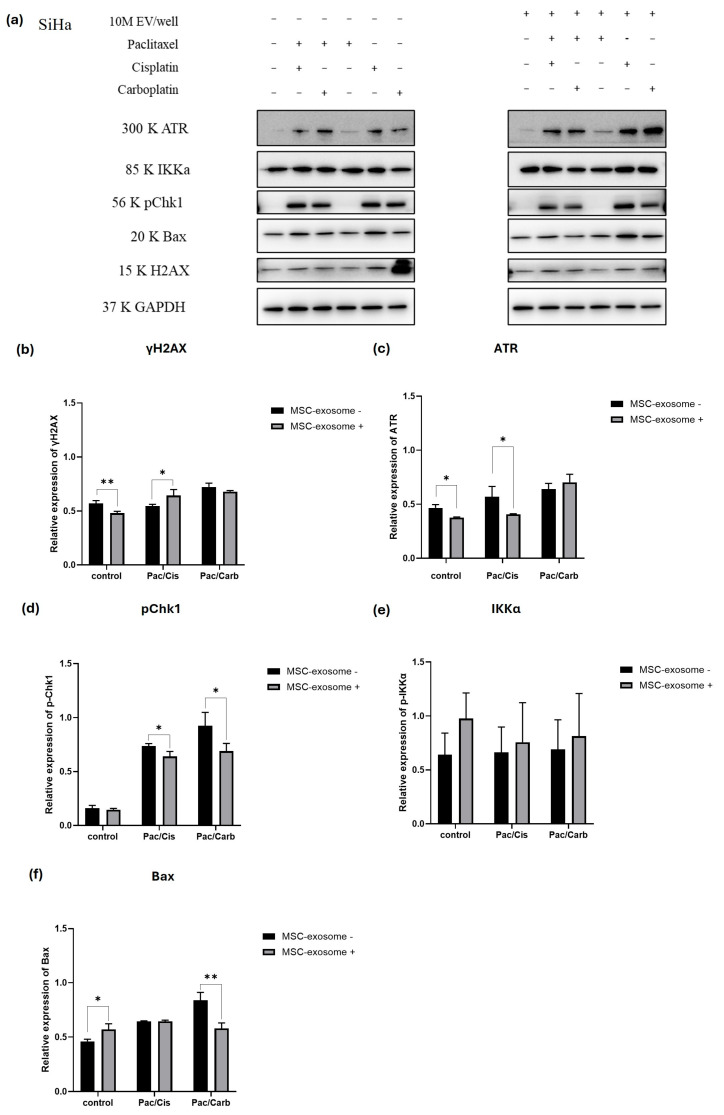
Western blot analysis of DNA damage, NF-κB, and apoptosis pathway proteins in SiHa spheroids following MSC-exosome pretreatment and combination chemotherapy. (**a**) Representative Western blot image showing protein expression profiles across SiHa spheroid treatment groups: untreated control, MSC-exosome pretreatment alone, and paclitaxel combined with either cisplatin or carboplatin, with or without MSC-exosome pretreatment. Densitometric analysis of (**b**) γH2AX, (**c**) ATR, (**d**) pChk1, (**e**) IKKα, and (**f**) Bax, with all expression levels normalized to those of GAPDH. Bar graphs represent mean ± SD from at least three independent experiments. Statistical significance was determined using Student’s *t*-test (* *p*-value < 0.05, ** *p*-value < 0.01).

We performed Western blot analysis targeting key regulators of the DNA damage response, apoptosis, and NF-κB signaling pathways to elucidate the molecular mechanisms underlying the differential responses of HeLa and SiHa spheroids to MSC-exosome pretreatment combined with chemotherapy. Specifically, we assessed the expression of selected markers, including ATR, pChk1, and γH2AX as markers of DNA damage; Bax as a pro-apoptotic protein; and IKKα and IκBα as central components of the NF-κB pathway, which promotes cell survival and therapy resistance. The inclusion of NF-κB signaling markers allowed us to evaluate the potential role of this pathway in mediating MSC-exosome-induced treatment effects. Protein expression was compared across treatment groups to determine how MSC-exosome pretreatment modulates downstream signaling in both HeLa and SiHa spheroids. Importantly, this analysis aimed to clarify whether MSC-exosomes contribute to chemotherapy sensitization or chemoresistance in the context of CC (Figure 8a and Figure 9a).

### 2.9. Alteration of gH2AX and IκBα Expression in MSC-EVs-Pretreated HeLa Spheroids Treated with Combination Chemotherapy

In addition to key apoptotic and survival markers, we evaluated γH2AX—the phosphorylated form of histone H2AX—which serves as a sensitive marker of DNA double-strand breaks. This protein was used to assess the extent of DNA damage induced by chemotherapy and the potential modulatory effects of MSC-exosome pretreatment. Compared to chemotherapy alone, the combination of MSC-exosome pretreatment and chemotherapy in HeLa spheroids led to a detectable increase in γH2AX expression. This finding supports the hypothesis that MSC-exosomes enhance chemotherapy-induced DNA damage, possibly by amplifying DNA damage signaling and impairing DNA repair mechanisms, thereby promoting apoptosis. Additionally, ATR and pChk1, both central to DNA damage response, displayed elevated expression in the combination treatment group, with the highest levels observed in spheroids pretreated with MSC-exosome and chemotherapy (Figure 8b–d). These results further indicate enhanced activation of the DNA damage checkpoint pathway following MSC-exosome priming. Regardless of MSC-exosome pretreatment, the expression of IKKα remained relatively unchanged across treatment groups (Figure 8e). However, IκBα levels were notably increased in the MSC-exosome plus chemotherapy group, which coincided with elevated γH2AX levels. Since IκBα is a well-established inhibitor of NF-κB signaling, its upregulation suggests a potential suppression of NF-κB activity, which may contribute to the observed increase in apoptosis and DNA damage (Figure 8f). This suppression of survival signaling was further supported by an elevated expression of Bax, which is a key pro-apoptotic protein. Bax levels exhibited an upward trend across all combination treatment groups, with the highest expression observed in spheroids pretreated with MSC-exosome (Figure 8g).

### 2.10. MSC-EV Pretreatment Combined with Dual Chemotherapy Modulates the DNA Damage Pathway in SiHa Spheroids

To investigate whether MSC-exosomes influence the DNA damage response and apoptotic pathways in CC cells, SiHa spheroids were treated with paclitaxel, cisplatin, or carboplatin, in combination with EVs (10 million EVs/well). The expression levels of ATR, IKKα, phosphorylated Chk1 (pChk1), Bax, and H2AX were analyzed by Western blotting, with GAPDH serving as the loading control. Treatment with paclitaxel, cisplatin, or carboplatin individually resulted in a detectable increasing trend in γH2AX, ATR and pChk1 expression compared with those in controls, which indicated activation of the DNA damage checkpoint pathway (Figure 9b–d). Combination treatment with MSC-exosomes further enhanced ATR and pChk1 expression, thereby suggesting that EVs may potentiate chemotherapeutic-induced DNA damage signaling. Similarly, pro-apoptotic related protein including Bax was upregulated following exposure to chemotherapeutic agents, with a more pronounced increase observed in exosome treatment groups. This may imply that MSC-exosomes augment the chemotherapy-induced apoptosis pathway. Moreover, γH2AX—a marker of DNA double-strand breaks—showed elevated expression upon drug treatment, which was further intensified in the presence of exosomes, thereby confirming that they enhanced DNA damage accumulation. In contrast, IKKα expression remained relatively constant across all treatments, which suggested that MSC-exosomes and chemotherapy primarily affected DNA damage and apoptotic pathways rather than NF-κB signaling (Figure 9e). Collectively, these results indicate that MSC-exosomes enhance the DNA damage response and apoptotic signaling triggered by paclitaxel, cisplatin, and carboplatin in SiHa cells, thereby potentially sensitizing CC cells to chemotherapeutic agents.

## 3. Discussion

Chemoresistance remains a principal barrier in CC treatment, with the tumor microenvironment playing a pivotal role in therapeutic outcomes. Our study leveraged 3D spheroid models of HeLa and SiHa cells—more physiologically relevant systems than traditional two-dimensional cultures—to examine how mesenchymal stem cell-derived exosome (MSC-exosome) pretreatment influences responses to combination chemotherapy. Using viability assays, stemness gene profiling, and pathway-specific Western blotting, we revealed that MSC-exosomes exert strikingly cell line-specific effects, such as sensitizing HeLa spheroids to a combination of chemotherapy while selectively modulating SiHa responses [13]. These results align with recent evidence highlighting the context-dependent roles of MSC-exosomes in CC spheroid models [14].

CSCs are a subpopulation of tumor cells characterized by their self-renewal capacity, pluripotency, and resistance to conventional therapies [14]. CSCs significantly contribute to tumor recurrence, metastasis, and treatment failure. In in vitro studies, 3D spheroid cultures better recapitulate the native tumor microenvironment than 2D conventional culture and are shown to enrich with CSCs-like phenotypes [15,16,17]. This concept is further reinforced by recent reviews highlighting that spheroid- and organoid-derived extracellular vesicles reflect tumor heterogeneity and microenvironmental pressures more accurately than 2D systems [18]. Aligning with this, our qRT-PCR analysis revealed significant upregulation of stemness-associated genes (OCT4, CXCR4, KLF4, and SOX2) in HeLa spheroids compared with their 2D counterparts, which is consistent with prior studies showing increased stemness in spheroid models [19,20,21,22]. Conversely, OCT4 expression was lower in SiHa spheroids than in 2D conventional cultures, suggesting a possible transition to a more differentiated phenotype [23,24,25,26]. This finding could reflect the heterogeneous nature of spheroids, where some cells retain stem-like traits while others differentiate. Additionally, the unique microenvironment of spheroids, characterized by hypoxia, nutrient gradients, and spatial limitations may suppress specific pluripotency genes (e.g., OCT4), while maintaining stress-related stemness markers. Notably, OCT4 is not the solitary determinant of stemness in all cancer types. Instead, other factors such as SOX2 and KLF4 may play a more important role in maintaining stemness phenotype. In CC, SOX2 and CD49f are often more highly expressed than OCT4 in both spheroid models and CSC-enriched populations [22,24,26]. Thus, the downregulation of OCT4 does not necessarily indicate a loss of stem-like properties but may represent an alternative “flavor” of stemness [25]. CD49f, a laminin-binding integrin, was highly expressed in both HeLa and SiHa spheroids which indicated enhanced interaction with the ECM and potential contribution to chemoresistance [27,28]. Moreover, the elevated expression of stemness-associated markers in both CC cell lines may underlie their distinct drug response profiles, highlighting the importance of targeting CSCs-related pathways in combination therapeutic strategies. Fluorescence microscopy confirmed efficient MSC-exosome internalization into both cell lines. Aligning with previous research on squamous head and neck cancers, fluorescence microscopy revealed that the green-labeled exosomes were predominantly localized within the cytoplasm and nucleus, which is consistent with true internalization rather than surface binding [29].

Our study showed that HeLa and SiHa CC cell lines responded differently to MSC-exosome treatment, thereby indicating a cell line-specific effect that warrants further investigation. Importantly, no clear dose-dependent cytotoxicity was observed during the chemotherapy dose optimization phase. For example, treatment with 10 and 50 µg/mL cisplatin induced widespread spheroid cell death, while treatment with 100 µg/mL preserved viable core cells, and treatment with 200 µg/mL primarily affected the peripheral cell population (Figure S1), with carboplatin exhibiting a similarly inconsistent response pattern (Figure S2). These irregularities suggest heterogeneous drug penetration or intrinsic resistance mechanisms within SiHa spheroids. Unlike the uniformly organized HeLa spheroids, SiHa spheroids tend to form looser, less cohesive structures, which may lead to uneven drug diffusion. This spatial heterogeneity likely contributed to region-specific responses within the spheroid, such as differential drug effects in the core vs. periphery, despite uniform dosing [30,31]. Moreover, the architectural features of SiHa spheroids, including densely packed cellular aggregates and limited interstitial spaces, may further hinder drug penetration and distribution. These structural limitations could contribute to the inconsistent chemotherapeutic responses observed. Further, the interaction between MSC-exosome and the tumor microenvironment may influence the expression of genes related to drug resistance, including those involved in the EMT and oxidative stress pathways. These findings underscore the multifactorial nature of chemoresistance in CC and highlight the potential of MSC-exosome as modulators of therapeutic response. Particularly, SiHa cells exhibit enhanced antioxidant defense mechanisms, with elevated expression of enzymes such as SOD1 and GPX1/2, which protect against ROS-induced cytotoxicity and contribute to their chemo-resistant phenotype [4,32].

Nonetheless, downstream responses diverged, thereby reflecting differences in intracellular signaling. Proteomic profiling of MSC-exosome via LC–MS/MS coupled with STRING network analysis revealed enrichment in ECM organization, focal adhesion, integrin–FAK–PI3K/AKT, and cytoskeletal pathways, which highlighted coordinated functional modules over isolated protein effects. The STRING PPI network complements the pathway enrichment results shown in the bubble plot, providing a detailed view of how the identified proteins are functionally interconnected. Consistent with the bubble plot, which highlighted ECM organization, focal adhesion, and integrin-mediated signaling as top chemotherapy-related pathways, the STRING network revealed a densely connected cluster of proteins within these same processes. Many of these proteins act as structural or regulatory “hubs,” including ECM remodeling enzymes, adhesion molecules, and cytoskeletal regulators, which play critical roles in controlling survival signaling in cancer cells. The integration of both analyses demonstrates that ECM remodeling and cytoskeleton-associated pathways are not isolated findings but represent central mechanisms underpinning chemoresistance and chemosensitivity. These observations align with recent literature highlighting the dual roles of MSC-derived small extracellular vesicles in regulating apoptosis, stress signaling, and therapeutic responsiveness [33].

Moreover, several protein groups were identified with functions in stress protection, including redox balance, inflammation control, and tissue repair. The HSP family acts as chaperones that maintain protein stability and promote cell survival under stress. Enzymes such as peroxiredoxins, thioredoxin-like protein, glutathione S-transferases, glutathione peroxidase, and biliverdin reductases, together with metabolic enzymes like G6PD, PGD, IDH1, and PHGDH, are crucial in antioxidant defense and ROS detoxification. Proteins involved in anti-inflammatory regulation, including annexins, TGFB1, S100 proteins, and AHSG, help modulate immune responses and protect tissues. Furthermore, ECM proteins such as fibronectin, vitronectin, collagens, laminins, integrins, SPARC, and thrombospondins contribute to wound healing, tissue remodeling, angiogenesis, and regeneration [34,35,36,37]. Collectively, these findings are consistent with those of other studies [34,35,36,37] and highlight that the proteins within the dataset encompass numerous protective mechanisms spanning anti-stress and antioxidant responses to anti-inflammatory effects and tissue repair, thereby contributing to enhanced cellular resilience under pathological or stress conditions in a particular HeLa spheroid. PKM2, an MSC-exosome protein, has been implicated in modulating drug sensitivity, thereby suggesting that exosome-mediated transfer of such proteins could possess dual roles of either promoting resistance or sensitization depending on the tumor context [38]. Moreover, our identification of apoptotic regulators such as ANXA1, ANXA2, LGALS1, and EEF2 underscores the potential of exosomes to modulate death signaling pathways [39,40]. Significantly, a pronounced cellular stress response was observed when HeLa cells were treated with cisplatin, carboplatin, paclitaxel, or combinations of these chemotherapeutic agents. This reflects the activation of survival pathways that often contribute to chemoresistance. Interestingly, pretreatment with MSC-exosome altered this response. Proteomic analysis revealed the presence of MSC-exosome-derived proteins, such as ANXA1 and EEF2, which appeared to suppress the activation of stress-related proteins within the target CC cells. This suppression not only reduced the cytoprotective stress response but also facilitated DNA repair mechanisms and enhanced the apoptotic cascade. These results suggest that MSC-exosomes may function as chemosensitizers by modulating cellular stress pathways to increase the susceptibility of HeLa spheroids to chemotherapy and thereby improving therapeutic efficacy.

The results obtained from HeLa and SiHa spheroids exhibited marked differences upon pretreatment with MSC-exosome. Despite both being CC cell lines, they exhibit distinct biological characteristics due to differences in their viral and genetic backgrounds. HeLa cells were derived from cervical carcinoma associated with HPV-18 and carry hundreds of copies of the viral genome that integrated into their DNA. This high copy number drives strong expression of viral oncogenes. This results in rapid proliferation and high metabolic activity, which makes HeLa cells highly suitable for studies of cell growth and immortalization. Meanwhile, SiHa cells originate from cervical carcinoma associated with HPV-16, which is generally linked to a more aggressive type than HeLa and invasive forms of CC. SiHa contains only one to two copies of integrated HPV DNA, but these integrations occur at critical sites in the host genome, subsequently profoundly disrupting cellular regulation. Consequently, SiHa cells show stronger activation of pathways associated with invasion, migration, and EMT, reflecting a more aggressive phenotype compared to that of HeLa. Therefore, while HeLa is characterized by extraordinary proliferative capacity, SiHa is more notable for its invasive and metastatic potential [41,42,43,44,45]. Both CC spheroids were formed in our previous study, and the marked difference in structure was observed. Thereafter, proteome profiles of HeLa and SiHa were analyzed. The results revealed that 8 of the top 20 expression proteins in SiHa spheroids exhibited association with chemotherapy resistance, particularly to cisplatin and paclitaxel [10]. For instance, S100A6, PGK1, PKM2, EEF2, HSP family, ANXA1, SLC3A2, and Rho GDI1 displayed associations with chemotherapy resistance. MSC-exosome proteins span processes such as drug efflux (ABC transporters), detoxification (glutathione metabolism), energy metabolism (glycolysis, Warburg effect, pentose phosphate pathway), and stress responses (UPR/ER stress, chaperone/proteasome systems). These findings align with those of previous reports that exosomes function not only as vehicles of intercellular communication but also as modulators of chemoresistance through the transfer of bioactive protein molecules, such as RNAs and metabolites [46]. One of the most prominent pathways enriched in the MSC-exosome was ECM remodeling, which included proteins such as FN1, COLs, and SPARC. ECM-related signaling has been widely linked to tumor cell adhesion, invasion, and drug penetration, all of which contribute to chemotherapy resistance [47]. Similarly, the integrin–FAK–PI3K/AKT signaling proteins (ITGAs, ITGBs, ILK, TLN1) identified in our dataset commonly regulate cell survival and drug resistance by promoting anti-apoptotic signaling [48]. Metabolic reprogramming was another hallmark as evidenced by the presence of PKM2, PGK1, LDHA, and related enzymes. These glycolytic proteins are associated with the Warburg effect, which enhances cancer cell survival under hypoxia and contributes to chemotherapy resistance [49,50].

Collectively, our results highlight that the MSC-exosome contains a repertoire of proteins that converge on pathways central to chemoresistance and, in certain contexts, chemosensitivity. These findings underscore the dual role of MSC-exosome: while they may promote therapy resistance via efflux, survival, and metabolic pathways, they may also offer therapeutic potential by delivering sensitizing signals or serving as biomarkers to guide drug selection. Future functional validation studies will be crucial to delineate the precise roles of individual exosomal proteins in modulating cisplatin, carboplatin, and paclitaxel responses in CC models.

The effect of MSC-exosome pretreatment on CC spheroids highlights the complexity of exosome-mediated modulation of chemotherapy sensitivity. Here, HeLa spheroids, which represent cervical adenocarcinoma, exhibited pronounced sensitization to both paclitaxel/cisplatin and paclitaxel/carboplatin regimens following MSC-exosome priming. This effect was evidenced by increased apoptotic activity, caspase activity, and reduced cell viability, thereby suggesting that MSC-exosome may carry pro-apoptotic or chemosensitizing molecules that enhance the efficacy of standard chemotherapeutic agents. Our Western blot analyses supported these observations by showing that MSC-exosome pretreatment enhanced chemotherapy-induced apoptosis in HeLa spheroids. This response was associated with the activation of pro-apoptotic signaling pathways, including the upregulation of Bax and DNA damage markers such as γH2AX and ATR. Bax promotes mitochondrial membrane permeabilization and the activation of cleaved caspase-3, which is a central executioner of apoptosis. Concurrently, MSC-exosome may downregulate anti-apoptotic proteins, thus facilitating apoptosis. The observed increase in cleaved caspase levels and red fluorescence in HeLa spheroids corroborates this response [13].

However, pretreatment of SiHa spheroids with MSC-exosomes selectively modulated their chemotherapeutic responses. Although the spheroids retained their inherent resistance to cisplatin, as indicated by sustained viability and limited apoptotic activity, they exhibited increased sensitivity to the combination of paclitaxel/carboplatin. This differential response suggests that MSC-exosome may influence specific cellular pathways, thereby potentially altering drug uptake, DNA repair mechanisms, or apoptotic signaling to sensitize SiHa cells to carboplatin. MSC-exosomes can carry regulatory proteins capable of modulating key molecular pathways. For instance, exosomal delivery of anti-apoptotic proteins may sensitize tumor cells to agents such as cisplatin and carboplatin. SiHa spheroids also displayed partial stemness characteristics, with significant upregulation of SOX2, downregulation of OCT4, and unchanged levels of KLF4/CXCR4 [51]. This selective activation of stemness-related genes may confer a survival advantage under stress conditions without full conversion to a CSCs phenotype. These structural and molecular differences may underlie the variability in drug responses observed in SiHa cells and highlight the importance of accounting for heterogeneity in 3D cancer models. Notably, SiHa spheroids—derived from HPV16-positive cervical squamous cell carcinoma—possess a notably aggressive nature and intrinsic resistance to chemotherapeutic agents, especially cisplatin. Here, MSC-exosome pretreatment did not improve sensitivity to paclitaxel/cisplatin but significantly enhanced responsiveness to the paclitaxel/carboplatin combination. This may reflect a differential susceptibility to carboplatin in SiHa cells potentially due to variations in drug uptake efficiency, DNA repair capacity, or apoptotic pathway activation influenced by MSC-exosome-carrying molecules. Cisplatin resistance in SiHa cells has been linked to a high expression of P16INK4A, which binds to CDK4 and inhibits the phosphorylation of retinoblastoma protein (pRb). This leads to cell cycle arrest and reduced apoptosis. Other intrinsic resistance mechanisms—such as activation of DNA repair pathways, expression of drug efflux pumps, and upregulation of survival pathways such as NF-κB—may vary across spheroid layers. Additionally, the presence of dormant cells in the spheroid core, inconsistent drug diffusion, adaptive stress responses, and microenvironmental conditions (e.g., hypoxia and nutrient gradients) likely contribute to the observed heterogeneity in treatment response [52,53,54]. Despite these differences, MSC-derived exosomes are promising potential enhancers of combination therapy. Beyond their endogenous modulatory effects, exosomes can be engineered to deliver therapeutic agents—including small RNAs and chemotherapeutics—directly to cancer cells [55,56]. Their biocompatibility, ability to cross biological barriers, and potential for targeted delivery make them attractive candidates for minimizing off-target effects and improving treatment specificity. Therefore, future studies should aim to characterize the molecular cargo of MSC-exosome to distinguish between tumor-suppressive and oncogenic components across various cancer subtypes.

Functionally, MSC-exosome treatment sensitized HeLa spheroids to paclitaxel/cisplatin and paclitaxel/carboplatin, with elevated caspase activity and DNA damage markers. Conversely, SiHa spheroids remained cisplatin-resistant but exhibited greater responsiveness to paclitaxel/carboplatin—likely due to their denser structure, limited drug penetration, and enhanced antioxidant defenses (SOD1, GPX1/2).

**Figure 10:**
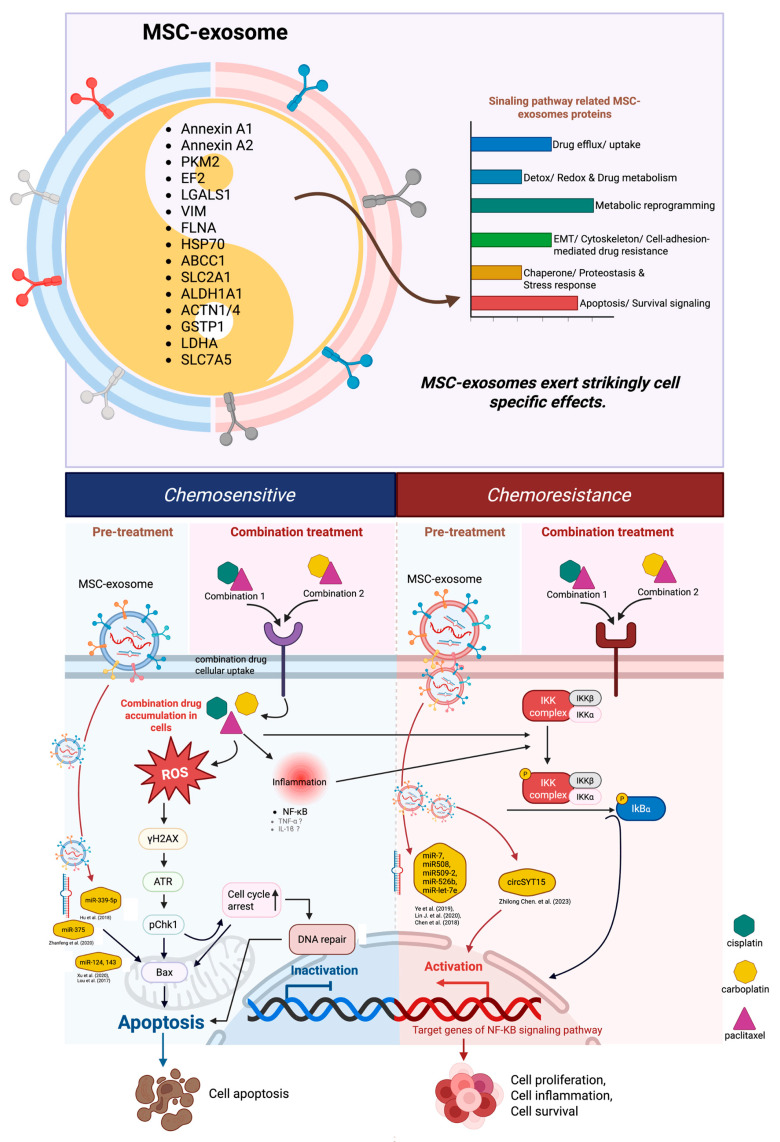
Schematic illustration of the proposed mechanism by which MSC-exosome pretreatment modulates combination chemotherapy responses in 3D CC spheroid models. The diagram highlights two contrasting roles of MSC-exosomes: left panel (blue): pro-apoptotic modulation, showing enhanced activation of apoptotic pathways and increased chemosensitivity; right panel (red): potential chemoresistance induction through the activation of survival pathways such as NF-κB and delivery of resistance-associated miRNAs. This model underscores the context-dependent dual role of MSC-exosomes in modulating therapeutic outcomes.

Collectively, our findings underscore the dualistic nature of MSC-exosome in CC therapy: they can promote resistance through detoxification and survival pathways yet also facilitate chemosensitization via cargo–mediated modulation. This highlights their potential as engineered adjuncts to therapy, contingent upon careful cargo characterization and tumor subtype profiling (Figure 10).

## 4. Materials and Methods

### 4.1. MSC Culture and Conditioned Medium Collection

MSCs were isolated from the bone marrow (BM) of healthy human donors and cultured in a defined medium. BM-MSCs were provided by Associate Professor Somchai Chutipongtanate (board-certified pediatrician, Ramathibodi Hospital, Mahidol University, Thailand) and the Environmental & Public Health Sciences Department at the University of Cincinnati College of Medicine, USA. Cells were maintained at 37 °C in a humidified atmosphere containing 5% CO_2_. The culture medium was replaced every 3–4 days. Cells were expanded in Dulbecco’s modified Eagle medium (DMEM) supplemented with 10% fetal bovine serum (FBS) and 1% penicillin/streptomycin (Invitrogen, Berlin, Germany) under standard incubation conditions. To deplete exosomes, FBS was ultracentrifuged at 110,000× *g* for 18 h at 4 °C using a Type MLA-50 rotor (Beckman Coulter, Brea, CA, USA). The resulting supernatant was used to prepare a 10% exosome-depleted FBS-containing medium, which had not previously contacted with cells and was used exclusively to collect MSC-derived exosomes.

MSCs were seeded at approximately 80% confluence before switching to the exosome-depleted medium for 72 h. The conditioned medium was then collected and centrifuged at 2500× *g* for 15 min to remove the cell debris, followed by centrifugation at 12,000× *g* for 45 min to eliminate the larger vesicles. The clarified supernatant was stored at −20 °C until further processing.

### 4.2. Isolation of MSC-Derived Exosomes from Conditioned Medium

MSC-derived exosomes were isolated and purified from the conditioned medium using a 10 kDa Amicon Ultra Centrifugal Filter (Sigma-Aldrich, St. Louis, MO, USA) followed by qEV size-exclusion chromatography columns (Izon Science, Christchurch, New Zealand), as per the manufacturer’s protocol. The collected fractions were pooled and subjected to ultracentrifugation at 110,000× *g* for 90 min at 4 °C using an Optima MAX-XP ultracentrifuge (Beckman Coulter) equipped with a TLA-110 fixed-angle rotor. Exosome pellets were resuspended in 1× phosphate-buffered saline (PBS, pH 7.4) and stored at −80 °C for subsequent analyses.

### 4.3. TEM

To assess morphology, purified MSC-derived exosomes were fixed with 2.5% glutaraldehyde and mounted onto Formvar-coated carbon grids. The grids were stained with 2% uranyl acetate, air-dried at room temperature, and visualized using a JEM-2010 transmission electron microscope (JEOL Ltd., Tokyo, Japan) [57].

### 4.4. NTA

MSC-derived exosomes were analyzed using a NanoSight NS300 Analyzer equipped with a 488 nm laser (Malvern Panalytical, Malvern, UK). Particle size and concentration were measured using NanoSight NTA software version 3.2, following previously described protocols [58]. Thawed MSC-derived exosomes were diluted in distilled water to a final volume of 1 mL. Two dilutions (1:1000 and 1:500) were prepared per sample to achieve particle concentrations of approximately 10^7^–10^9^ particles/mL (corresponding to 20–100 particles/frame). Data were acquired at a camera level of 12 and a detection threshold of 6 over five 30 s video captures per sample. The absolute particle concentrations for each dilution were calculated using the respective dilution factors (1:1000 and 1:500). Statistical analysis was performed using GraphPad Prism version 10.6.1 (GraphPad Software, San Diego, CA, USA). Particle concentrations (10^9^ particles/mL) and size distributions are reported as medians with interquartile ranges. The Shapiro–Wilk test was used to assess normality, and group differences were analyzed using the Mann–Whitney U test. Statistical significance was set at *p* ≤ 0.05.

### 4.5. Protein Digestion

Triplicate of MSC-derived exosomes was digested using a protein aggregation capture protocol [59]. Briefly, 25 μg of each sample was diluted in PBS with 2% SDS and then heated at 95 °C for 10 min. The samples were subsequently incubated in a dark room with 20 mM TCEP and 10 mM CAA at room temperature for 30 min. Next, 125 μg of MagReSyn^®^ Hydroxyl beads (ResynBio, Gauteng, South Africa) were added to each sample. The final working volume was adjusted to 50 μL before inducing protein aggregation by increasing the acetonitrile (ACN) concentration to 70%. The aggregated proteins were washed twice with 80% ethanol and once with 100% ACN. On-bead digestion was performed using trypsin at a ratio of 50:1 (protein:enzyme, *w*/*w*) in TEAB buffer, followed by incubation at 37 °C overnight. Then, the digested samples were acidified with 1% formic acid prior to LC-MS/MS analysis.

### 4.6. LC-MS/MS Analysis

Sample analysis was performed using a Vanquish Neo UHPLC system (Thermo Fisher Scientific, Waltham, MA, USA) coupled to an Orbitrap Exploris 480 mass spectrometer (Thermo Fisher Scientific, Waltham, MA, USA). A total of 200 µg of tryptic digest was injected onto an Acclaim PepMap C18 trap column (3 μm, 75 μm × 20 mm; Thermo Fisher Scientific, Waltham, MA, USA). Peptide separation was carried out using a PepMap C18 analytical column (2 μm, 75 μm × 500 mm; Thermo Fisher Scientific, Waltham, MA, USA) at a flow rate of 0.3 μL/min and an electrospray voltage of 2 kV and a 60 °C column temperature. Subsequently, a linear gradient was applied from 2% to 45% solvent B (solvent A: 2% acetonitrile + 0.1% formic acid; solvent B: 80% acetonitrile + 0.1% formic acid) over 90 min, followed by a 10 min wash at 95% solvent B. MS1 scans were in the *m*/*z* range of 400–1000 at 120,000 resolution and an AGC target of 3 × 106. MS2 spectra were acquired using data-independent acquisition at 20 *m*/*z* isolation windows, 30,000 resolutions, an AGC target of 2 × 106, and a normalized collision energy (NCE) of 28% using higher-energy collisional dissociation. The RF lens was set to 50%.

### 4.7. Proteomic Data Processing

Raw data from data-independent acquisition (DIA) experiments were analyzed using DIA-NN version 2.2.0 [59], searched against predicted spectra from the UniProt Homo sapiens proteome (UP000005640) with the following parameters: one missed cleavage, N-terminal excision, carbamidomethylation fixed modification, generic scoring, MS1 and MS2 mass tolerance set to 10 ppm and 10 ppm, respectively, QuantUMS quantification, retention time-dependent normalization, and enabled match-between-runs functionality. Precursor and protein identifications were filtered at a false discovery rate (FDR) <0.01.

### 4.8. Molecular and Functional Analysis

Proteins identified in more than 50% of biological replicates were retained for downstream analysis. Functional enrichment analyses were performed to characterize the biological relevance of the identified proteins. Gene ontology (GO) enrichment for the biological process and molecular function categories was conducted using the PANTHER database (http://www.pantherdb.org; accessed on 1 September 2025). Pathway enrichment analysis was additionally performed using the Reactome database (accessed on 1 September 2025) to identify chemotherapy-related pathways. All pathway analysis was generated using FDR < 0.05. R version 4.5.1 (R foundation for Statistical Computing, Vienna, Austria) was used to generate the bubble plot via ggplot2, dplyr, stringr, and forcats packages. To visualize the PPI cluster, STRING database was used (Version 12.0; https://string-db.org, accessed on September 2025). The clusters are identified using computational algorithms. We used k-means clustering as a clustering option and set it to 10 clusters based on their centroids. Each cluster was represented in a different color.

### 4.9. CC Cell Culture

The CC cell lines used in this study were HeLa (HPV–18–positive adenocarcinoma, ATCC CCL–2) and SiHa (HPV–16–positive squamous cell carcinoma, ATCC HTB–35), with both being obtained from the American Type Culture Collection (ATCC, Manassas, VA, USA). Cells were cultured in DMEM supplemented with 10% FBS and 1% penicillin/streptomycin (Invitrogen, Carlsbad, CA, USA) at 37 °C in a humidified atmosphere containing 5% CO_2_ [10].

### 4.10. Spheroid Formation in Poly-HEMA-Coated Plates

A 50 mg/mL solution of poly-HEMA (Sigma-Aldrich, St. Louis, MO, USA) was prepared in ethanol by stirring with a magnetic bar for 8 h at room temperature. Thereafter, 15–20 µL of the solution was added to each well of a 96-well U-bottom plate, and the plate was incubated overnight to dry and form a non-adhesive coating. For spheroid formation, 5000 CC cells suspended in 150 μL of complete medium were seeded into each well of the coated 96-well plate. Plates were incubated at 37 °C in a 5% CO_2_ atmosphere. HeLa cells formed spheroids within 3 days, while SiHa required seven days. The medium was replaced every 72 h during spheroid formation.

### 4.11. Characterization of Spheroid Formation

Spheroid size was initially evaluated using an inverted microscope and analyzed using ImageJ software (version 1.54). Cell viability was assessed using a live/dead^®^ cell imaging kit (Thermo Fisher Scientific, Waltham, MA, USA), which includes calcein AM to label live cells and BOBO-3 iodide to label dead cells. Phase-contrast and fluorescence images were captured using a Lionheart FX live-cell imaging system (Agilent Technologies, Santa Clara, CA, USA).

An immunofluorescence assay was performed to assess the expression of the CD49f surface marker. Spheroids were fixed in 4% paraformaldehyde for 20 min and incubated with phycoerythrin (PE)-conjugated anti-human CD44 antibody (ImmunoTools GmbH, Friesoythe, Germany) for 45 min. Nuclei were counterstained with DAPI (Sigma-Aldrich), and fluorescent images were acquired using a Lionheart FX live-cell imaging system.

For gene expression analysis, total RNA was extracted from both monolayer and spheroid cultures using TRIzol reagent (Invitrogen, Carlsbad, CA, USA), according to the manufacturer’s instructions. RNA concentration and purity were measured using a NanoDrop spectrophotometer (Thermo Fisher Scientific, Waltham, MA, USA). One microgram of high-quality RNA was reverse transcribed into complementary DNA (cDNA) using a cDNA synthesis kit (Bio-Rad, Hercules, CA, USA), according to the manufacturer’s protocol. The relative expression of stemness-related genes—including OCT4, SOX2, CXCR4, and KLF4—was analyzed. Primer sequences are listed in Table S1. qRT-PCR was performed in duplicate across three independent experiments. Relative expression levels were calculated using the 2-∆Ct method. The housekeeping gene, GAPDH, was used as an internal control.

### 4.12. pKH Labeling of MSC-Derived Exosomes and Uptake Assay

To examine exosome uptake by CC cells, MSC-derived exosomes were labeled with PKH67 dye (Sigma-Aldrich, St. Louis, MO, USA). Briefly, 50 μg of exosomal protein was diluted in 1 mL of 1X PBS and mixed with 4 μL of PKH67 dye dissolved in 1 mL of Diluent C. The mixture was incubated at room temperature for 5 min to allow labeling. To stop the labeling reaction, bovine serum albumin was added. Labeled MSC-derived exosomes were re-isolated via ultracentrifugation at 110,000× *g* for 90 min. The resulting exosome pellets were resuspended in 1X PBS and stored at −80 °C for further analysis.

### 4.13. Single and Combination Chemotherapy

HeLa and SiHa cells grown in poly-HEMA-coated plates were treated with single chemotherapeutic agents—cisplatin, carboplatin, or paclitaxel—at concentrations of 10, 50, 100, and 200 μg/mL for 72 h. Post-treatment, spheroid morphology, size, and cell viability were evaluated. Spheroid morphology was visualized using an inverted microscope, and spheroid size was measured and analyzed using ImageJ software. Cell viability was assessed using a Live/DEAD^®^ Cell Imaging Kit (Thermo Fisher Scientific, Waltham, MA, USA). For staining, the culture medium was removed from each well, while leaving approximately 50 μL. The Live/DEAD staining solution was then added. Plates were wrapped in foil to protect them from light and incubated at 37 °C for 30 min. Fluorescent images were captured using a Lionheart FX live-cell imaging system. In addition to qualitative imaging, cell viability was quantitatively measured using the CellTiter-Glo^®^ 3D cell viability assay (Promega, Beijing, China). The IC_50_ values for cisplatin and carboplatin were calculated from dose–response curves. Subsequently, these IC_50_ values were used to determine appropriate drug concentrations for the combination chemotherapy experiments.

### 4.14. MSC-Exosome Pretreatment Followed by Combination Chemotherapy in CC Spheroids

Spheroids were pretreated with MSC-derived exosomes at a dose of 10 million particles per well (equivalent to 1.56 µg of BM-MSC-derived exosomal protein) for 24 h prior to chemotherapy administration. Following pretreatment, spheroids were treated with paclitaxel in combination with either cisplatin or carboplatin, diluted in complete DMEM. For HeLa spheroids, the final concentrations of cisplatin and carboplatin were 3.025 µg/mL and 24.545 µg/mL, respectively. For SiHa spheroids, the respective concentrations were 8.725 µg/mL and 78.50 µg/mL. After 72 h of combination treatment, spheroids were assessed for cell viability, caspase activity, and molecular pathway activation to evaluate treatment response.

### 4.15. Cell Viability and Caspase Activity Assay

Cell viability was quantified using the CellTiter-Glo^®^ 3D cell viability assay, which measures intracellular ATP as an indicator of metabolically active cells. The CellTiter-Glo^®^ 3D reagent was added to each well in a volume equal to the existing culture medium, mixed thoroughly to induce cell lysis, and incubated at room temperature for 25 min to stabilize the luminescent signal.

The ApoLive-Glo^TM^ multiplex assay(Promega Corporation, Madison, WI, USA) ) was used to measure both live-cell ATP levels and caspase-3/7 activity in 3D CC spheroids to simultaneously assess cell viability and apoptosis—operationally defined as caspase-3/7 activation. This assay is particularly well suited for 3D spheroid models, where conventional apoptosis assays are often limited by spheroid architecture and reagent penetration. Following MSC-exosome pretreatment and subsequent combination chemotherapy, SiHa and HeLa spheroids were incubated with the ApoLive-Glo™ reagent (containing both viability and caspase 3/7 substrates), according to the manufacturer’s instructions. After 30 min of incubation at 37 °C, fluorescence (excitation/emission: 400 nm/505 nm) was measured using a Varioskan^Tm^ LUX Multimode Microplate Reader (Thermo Fisher Scientific, Waltham, MA, USA) to assess cell viability. Subsequently, 20 µL of caspase-Glo^®^ 3/7 reagent was added to each well, gently mixed, and incubated for an additional 30 min at room temperature in the dark. Luminescence was recorded to determine caspase-3/7 activity. All measurements were performed in triplicate. This multiplex approach enabled the simultaneous evaluation of cytotoxicity and apoptotic activation in response to MSC-exosome pretreatment and combination chemotherapy.

### 4.16. Western Blot Analysis

Treated spheroid pellets were lysed using 1X RIPA buffer (Pierce Biotechnology, Rockford, IL, USA). Lysates were incubated on ice for 20 min followed by centrifugation at 12,000× *g* to remove debris. Protein concentrations were determined using the Bradford assay (Bio-Rad Laboratories, Hercules, CA, USA). A total of 20 µg of protein per sample was separated on 12% SDS-PAGE gels and transferred to polyvinylidene difluoride membranes (Amersham Pharmacia Biotech, Piscataway, NJ, USA). Membranes were blocked with 5% non-fat dry milk in Tris-buffered saline containing 0.1% Tween-20 (TBS-T) for 1 h at room temperature. After blocking, membranes were washed twice with TBS-T for 10 min each and incubated overnight at 4 °C with primary antibodies diluted at 1:1000 in 1% non-fat milk in TBS-T. For EV characterization, tetraspanin surface markers, including CD63 (D4I1X, 1:500) and CD9 (D801A, 1:500) from Cell Signaling Technology (Danvers, MA, USA) were used. β-actin served as the internal loading control. To investigate molecular mechanisms, primary antibodies targeting three key signaling pathways—DNA damage response, NF-κB, and apoptosis—were used (see Table S2 for antibody details). After primary antibody incubation, membranes were washed thrice with TBS-T (10 min each), followed by a 2 h incubation with a secondary antibody (anti-rabbit IgG horseradish peroxidase; cat. no. 7074, Cell Signaling Technology, Danvers, MA, USA) diluted at 1:2000 in 1% non-fat milk in TBS-T. Protein expression was detected using enhanced chemiluminescence (Pierce™ ECL Western blotting Substrate, Thermo Fisher Scientific, Waltham, MA, USA). Signal intensities were measured using chemiluminescence imaging by using an Epi Fluorescence Alliance Q9 Advanced Imager (UVITEC Ltd., Cambridge, UK).

## 5. Conclusions

Our findings underscore the dualistic nature of MSC-exosome in CC therapy: they can promote resistance through detoxification and survival pathways, yet also facilitate chemosensitization via cargo-mediated modulation. This highlights their potential as engineered adjuncts to therapy, contingent upon careful cargo characterization and tumor subtype profiling.

## Figures and Tables

**Table 1:** IC_50_ values of single chemotherapeutic agents against cervical cancer spheroids.

Spheroid Type	Cisplatin (µg/mL, Mean ± SD)	Carboplatin (µg/mL, Mean ± SD)
HeLa	6.05 ± 0.52	49.09 ± 0.61
SiHa	17.45 ± 0.49	157.01 ± 10.03

**Table 2:** MSC-exosome proteins and associated functional pathways.

Pathway/Process-Related	MSC-Exosome Proteins
ABC transport/Drug efflux	ABCC1, ABCC4
Glutathione/Detox	GSTP1, GSTT1; GSR/GPX3/PRDX1/2/4/6, TXNL1
Aldo–keto reductases/Aldehyde dehydrogenases	AKR1B1, AKR1B10, AKR1C1/3, ALDH1A1/ALDH9A1
Glycolysis/Warburg	PKM (PKM2), PGK1, LDHA/LDHB, ENO1, PFKP/PFKL, TPI1, PGAM1, ALDOA/ALDOC, FASN/ACLY
PPP (Pentose phosphate)	G6PD, PGD, TALDO1, TKT
One-carbon/Folate	DHFR/DHFR2, MTHFD1, ALDH1L1
Lactate export/pH	SLC16A3 (MCT4)
Amino acid transport/mTOR fueling	SLC7A5, SLC1A5
EMT/Cytoskeleton	VIM, FLNA, FSCN1, ACTN1/4, IQGAP1, EZR, TAGLN
Integrin–FAK–PI3K/AKT/Focal adhesion	ITGA1/2/3/4/5/6/11, ITGAV, ITGB1/2/3/5, ILK, TLN1, LIMS1, VCL, ZYX
ECM remodeling/Barrier	FN1, COLs, SPARC, POSTN, TNC, THBS1/2/4, LOX/LOXL2/3, VCAN, TGFBI
RTK/MAPK/PI3K-AKT	MAPK1, PDGFRB, PRKCB, YES1/LYN, PIK3CG, STAT1
Ras/Rho family	KRAS/NRAS, RAC1, CDC42, ARHGDIA (RhoGDI1), RALA/RALB
Chaperone/Proteostasis	HSP90AA1/HSP90AB1/HSP90B1, HSPA1A/B/HSPA2/4/5/8/13, ST13, VCP, UBA1/UBE2/PSMA/PSMC/PSMD
UPR/ER stress	HSPA5 (GRP78), PDIA3, HSP90B1
Autophagy/Mitophagy	MAP1LC3A/B/B2 (LC3), DNM1L
Exosome/Endo-Exocytosis (drug export/signaling)	RAB27A/B, RAB4/5/7/8/10/11/13/14/18/21/23/31/32/35, SNAP23, STX4/7, STXBP2/3, TSG101, PDCD6IP (ALIX), SCAMP3, TOM1
Apoptosis/Death signaling	ANXA2, LGALS1, ANXA1, EEF2
TGF-β/NOTCH	TGFB1, TGFBI, NOTCH2/NOTCH3
Caveolae/Endocytosis	CAV1/CAVIN1
Vault-mediated transport	MVP
Anti-stress (Chaperones)	HSP90AA1, HSP90AB1, HSP90B1, HSPA1A/B, HSPA5, HSPA8, HSPB1, HSPB6, HSPA2, HSPA4
Anti-ROS/Redox balance	PRDX1, PRDX2, PRDX4, PRDX6; TXNL1; GSTP1, GSTT1/2, GSTM2; GPX3; CYB5R3; BLVRB, BLVRA; G6PD, PGD; IDH1; PHGDH
Anti-inflammation/Immune modulation	ANXA1, ANXA2, ANXA5, ANXA6; TGFB1; S100A6, S100A10, S100A11; AHSG
Healing/Wound repair (ECM and adhesion)	FN1, VTN, SPARC, DCN, TNC; Collagens (COL1A1, COL1A2, COL3A1, COL4A1/2, COL5A1/2/3, COL6A1-3, COL18A1); Laminins (LAMA1/2/4, LAMB1/2, LAMC1); Integrins (ITGB1, ITGB3, ITGB5, ITGA5, ITGA6); THBS1, THBS2, THBS4

## Data Availability

The data presented in this study are available on request from the corresponding author. The data are not publicly available due to restrictions imposed by collaborative research agreements.

## Supplementary Materials

The following supporting information can be downloaded at: https://www.mdpi.com/article/10.3390/ijms27031575/s1.

## Abbreviations

The following abbreviations are used in this manuscript:

CC	Cervical cancer
MSC-exosome	Mesenchymal stem cell-derived exosome
3D	Three-dimensional
NTA	Nanoparticle tracking analysis
TEM	Transmission electron microscopy
CSC	Cancer stem cell
ECM	Extracellular matrix
OCT4	Octamer-binding transcription factor 4
CXCR4	C-X-C chemokine receptor type 4
KLF4	Krüppel-like factor 4
SOX2	SRY-box transcription factor 2
BP	Biological processes
MF	Molecular functions
PPI	Protein–protein interaction
HSPs	Heat shock proteins
ROS	Reactive oxygen species
FN1	Fibronectin
DCN	Decorin
TNC	Tenascin-C
VTN	Vitronectin
EMT	Epithelial–mesenchymal transition
BM	Bone marrow
DMEM	Dulbecco’s modified Eagle medium
FBS	Fetal bovine serum
ACN	Acetonitrile
FDR	False discovery rate
GO	Gene ontology
DAPI	4′,6-diamidino-2-phenylindole dihydrochloride
PE	Phycoerythrin
cDNA	Complementary DNA
qRT-PCR	Quantitative reverse transcription PCR
TBS-T	Tris-buffered saline containing 0.1% Tween-20
EV	Extracellular vesicle
