# Supplementary material for: Mesenchymal Stem Cell-Derived Exosomes Reprogram Chemosensitivity Pathways in Cervical Cancer Spheroids
Source: Int J Mol Sci. 2026 Feb 5;27(3):1575. doi: 10.3390/ijms27031575 (PMC12898660; doi:10.3390/ijms27031575)
Supplement: Supplementary file 1 [file ijms-27-01575-s001.zip › Supplementary_Figures.pdf]

Supplementary Figures

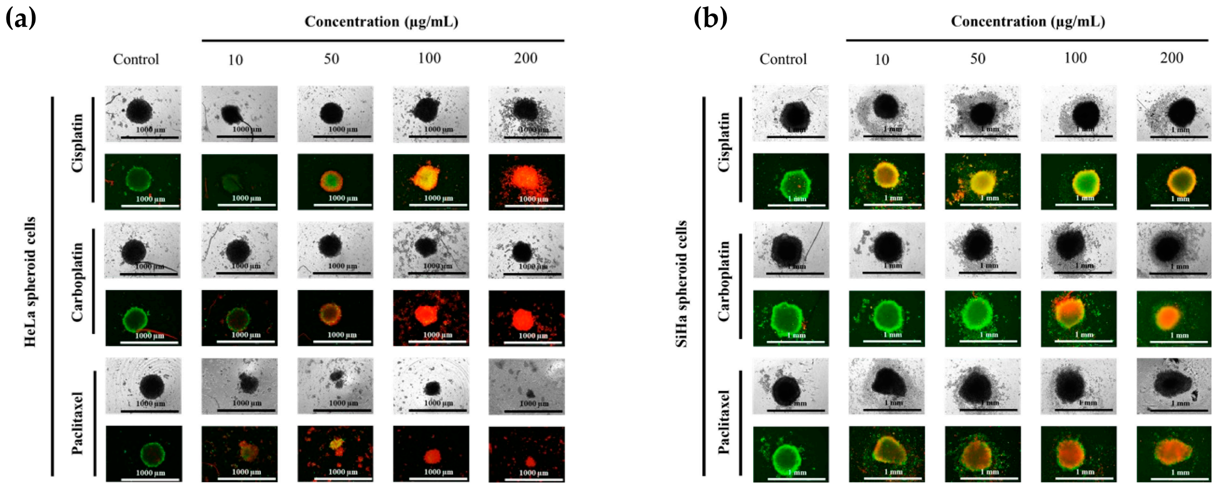

Figure S1. Dose-Response Effects of Cisplatin, Carboplatin, and Paclitaxel on HeLa and SiHa Spheroids visualized via Live/Dead staining. CC spheroids were treated with increasing concentrations of individual chemotherapeutic agents. Live cells were stained with calcein AM (green fluorescence), while dead cells were labeled with ethidium homodimer-1 (red fluorescence). Yellow fluorescence indicated overlapping signals from live and dead cells.

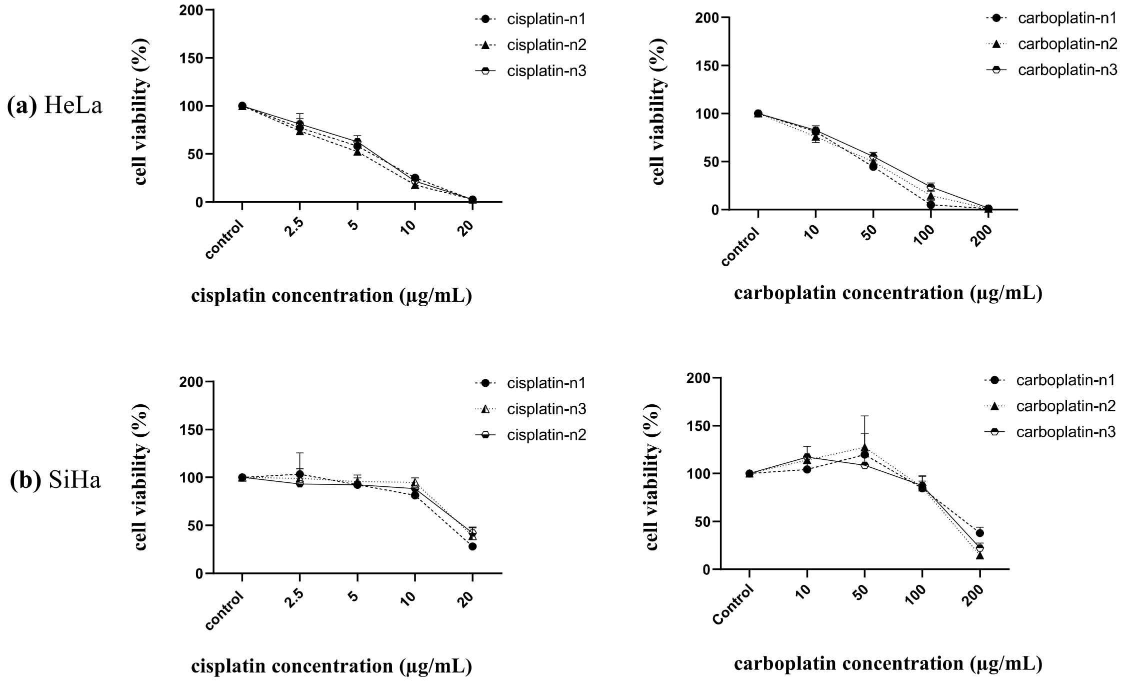

Figure S2. Optimization of single-agent chemotherapy doses on CC spheroids. **(a)** HeLa and **(b)** SiHa spheroids were treated with varying concentrations of cisplatin and carboplatin. Cell viability was quantified using a CellTiter-Glo assay. Data are presented as mean ± SD from three independent experiments.
