# Supplementary material for: Mesenchymal Stem Cell-Derived Exosomes Reprogram Chemosensitivity Pathways in Cervical Cancer Spheroids
Source: Int J Mol Sci. 2026 Feb 5;27(3):1575. doi: 10.3390/ijms27031575 (PMC12898660; doi:10.3390/ijms27031575)
Supplement: Supplementary file 1 [file ijms-27-01575-s001.zip › Supplementary_Tables.pdf]

Supplementary Tables

Table S1. Primer sequences used for stemness gene expression analysis.

Gene	Sequence (5'–3')	Gene groups
GAPDH-F	ACG GAT TTG GTC GTA TTG G	Housekeeping gene
GAPDH-R	GGA AGA TGG TGA TGG GAT TT	
SOX2-F	CTC CGG GAC ATG ATC AGC	Stemness markers
SOX2-R	GGT AGT GCT GGG ACA TGT G	
OCT4-F	CCC AAT TAC CCA TCC TTC CTG	
OCT4-R	GTC TTC CCC TCT TTG GCT TG	
KLF4-F	CTG GAG AAG GAG AAG CTG GA	
KLF4-R	CAA ATT GCT CGA GTT CTT TCT G	
CXCR4-F	CCC TCA AGA CCA CAG TCA TCC	
CXCR4-R	GTT CTC AAA CTC ACA CCC TTG C	

Table S2. Antibodies used in this study.

Antibody (Cat No.)	Molecular weight (kDa)	Dilution
**Antibodies for BM-MSCs-Exo**		
• CD9 Rabbit mAb (13174)	22–24	1:500
• CD63 Rabbit mAb (52090)	25–60	1:500
• β-Actin Antibody (4967)	45	1:1,000
• Cytochrome c Rabbit mAb (11940)	14	1:1,000
**Antibodies for molecular pathway analysis**		
o DNA damage pathway		
• Phospho-ATR	300	1:1,000
• Phospho-Chk1 (Ser345) Rabbit mAb (2348)	56	1:1,000
• Phospho-Histone H2A.X (Ser139) Rabbit mAb (9718)	15	1:1,000
o NF-κB Pathway		
• Phospho-IKKα/β (Ser176/180) Rabbit mAb (2697)	85	1:1,000
• Phospho-IκBα (Ser32) Rabbit mAb (2859)	40	1:1,000
o Apoptosis pathway		
• Bax (D2E11) Rabbit mAb (5023)	20	1:1,000
**Internal control**		
• GAPDH Rabbit mAb (2118)	37	1:1,000
**Secondary antibody**		
• Anti-rabbit IgG, HRP-linked Antibody (7074)		1:1,000
